# Effects of financial compensation structures on community health worker performance, motivation, and retention: evidence from a multi-arm quasi-experiment in Uganda

**DOI:** 10.3389/frhs.2025.1687782

**Published:** 2025-12-11

**Authors:** Moses Okech, Tabither Muthoni Gitau, Wilfred Zoungrana, Erick Kiprotich Yegon, Nzomo Mwita, Stella Kanyerere, Alice Koimur, Arthur Arinda, Kabanda Richard, Ruth Chitwa

**Affiliations:** 1 Living Goods, Nairobi, Kenya; 2 Community Health Department, Republic of Uganda Ministry of Health, Kampala, Uganda

**Keywords:** community health workers, compensation, performance-based financing, supportive supervision, motivation, retention, primary healthcare, Uganda

## Abstract

Community Health Workers (CHWs) are central to extending primary health care in low-resource settings, yet their compensation remains a policy challenge. This paper reports findings from a five-arm quasi-experimental study conducted by Living Goods Uganda to test how different mixes of fixed stipends and performance-based incentives (PBIs) affect CHW performance, motivation, and retention. Over a nine-month period, 1,104 CHWs were assigned to five compensation models—70:30, 50:50, 30:70 PBI-to-stipend ratios, a 100% stipend arm, and a control—implemented across five districts. Quantitative data were analyzed using a difference-in-differences model with cluster-robust standard errors and wild-bootstrap inference, complemented by qualitative interviews and focus groups exploring experiences and perceptions. Results showed that moderate performance-based incentives (30%–50%) achieved the most balanced outcomes: improved household coverage, immunization follow-up, and referrals, alongside higher motivation and satisfaction. The 70% PBI arm generated stronger performance gains but increased stress and reduced retention, while the stipend-only arm offered stability but lower service coverage. Overall retention exceeded 95%, though sustained motivation depended heavily on supervision quality, recognition, and fairness of pay. Findings highlight that hybrid pay structures combining predictable stipends with moderate PBIs can enhance CHW productivity while safeguarding motivation and sustainability. The study offers practical guidance for Uganda's National Community Health Strategy and similar programs seeking equitable, gender-sensitive, and financially feasible CHW compensation models.

## 1 Introduction

CHWs are acknowledged as a heterogenous cadre of persons (such as receiving a few days to a few months of basic training, and having varied literacy levels), deployed at various levels of the health systems, mostly within communities they serve, but sometimes spending significant durations at primary care health facilities. Also, CHWs are characterized by having in-depth knowledge of their communities, thereby providing culturally appropriate health services. A systematic review of definitions including 119 empirical research articles from 25 countries across all regions globally defines a Community Health Worker (CHW) as “*a cadre of lay health workers who provide health promotion and disease prevention services in their community”* (1). Typically, the CHWs tend to provide basic health services as unpaid volunteers, often receiving allowances for completed tasks, with a few para-professionals cadres being salaried (1, 2).

Globally, community health worker (CHW) compensation models span a wide continuum—from volunteer-based systems to fully salaried cadres—with distinct implications for motivation, performance, and sustainability. Empirical studies from Sierra Leone, Kenya, and India demonstrate how the structure of financial incentives shapes CHW recruitment, behavior, and retention. In Sierra Leone, Deserranno et al. (3) found that higher pay attracted more capable but extrinsically motivated CHWs, influencing both selection and performance. In Kenya, Brunie et al. (4) observed that performance-based incentives improved short-term outputs but increased stress and data inflation, underscoring the need for balanced mixes. India's ASHA program highlights that heavy reliance on task-based incentives can demotivate CHWs when payments are delayed, leading to task selectivity (5). Global syntheses further show that predictable and fair pay is often valued as highly as incentive size (6, 7). Building on this evidence, the present quasi-experiment extends prior single-model studies by systematically varying the ratio of fixed stipend to performance-based incentives across multiple sites in Uganda.

Learning from exemplar programs that have adapted PHC models, which are prevalent in several low- and middle- income countries (LMICs), characterized by decentralized health services delivery, and building nationally adaptive CHW programs. Several countries have moved the direction of mainly a voluntary CHW workforce cadre (2, 8) raising concerns around sustainability, the cost and value of CHW programs, but a few adapting a paid/ salaried cadre e.g., (Ethiopia), while others have developed a blended model having both formally employed and paid cadres working along the volunteer CHWs, e.g., South Africa (23). In sum, health economists and the WHO have signaled that voluntary CHW national programs appear inefficient because of poor performance (9), and may be unsustainable (10, 24).

At the dawn of the Sustainable Development Goals (SDG) era in 2016, it became apparent as exemplified in the Workforce 2030 strategy that the global skilled health workforce was in shortage by more than 18 million cadres, a situation poised to more than double by 2030 (10). Therefore, for achieving UHC, governments were being urged to seek alternatives to the skilled health workforce, such as task sharing, task shifting to lower-level cadres, and eventually to also grow the national CHW programs. However, the non-compensation of CHWs, alongside weak supervision and overall weak governance of CHWs increasing attrition (World Bank group series, 2021) are known to undermine the performance of CHW programs, as evidenced from the Integrated Community Case Management (ICCM) of childhood illnesses programs (11).

In response, the (WHO provided guidelines for strengthening the country CHW programs (12), suggesting among other levers, the implementation of a combination of financial and non-financial incentives. Recommendations were based on the existing evidence from discrete choice experiments (DCEs) exploring CHW work preferences in contexts such as Bangladesh, Haiti, Kenya and Uganda (2, 13), and from systematic reviews (7, 14). In Uganda and Kenya, higher compensation, reliable personal transport to aid work such as bicycles, mobile phones and recognition were prioritized by CHWs (2, 4, 13).

In Uganda, most CHWs (Village Health Teams—VHTs) are volunteers, which has raised concerns about motivation, workload, and sustainability. Studies show that only one in five CHWs performs optimally on integrated community case management (iCCM) tasks such as under-five diagnosis, referral, and home visits (15, 16). Female gender, secondary level education or higher, availability of iCCM drugs and supplies, community support, and supervisory support were positively associated with performance, while high workload was negatively associated, which findings resonate with the known CHW motivators globally (17).

Learning from performance-based financing strategies employed for improving the performance of the skilled health workforce (18–20), the evidence points to process improvements such as for shorter waiting times for clients, better client satisfaction with consulting visits, more efficient triage, and better quality of health education. However, there is less evidence on the effectiveness for improving patient-related outcomes such as clinical effectiveness (timely diagnosis, appropriate treatments and higher cure rates), and for system related outcomes such as better quality of care, better access to, and coverage of care services. Therefore, performance-based incentive approaches appear to be effective especially within quality—improvement systems for improving care processes. Though performance-based financing (PBF) schemes tend to be complex interventions, tagging multiple indicators for team, institutional, and system level outcomes, therefore, more context specific evidence is required to unpack the scenarios within which PBF is premised to work for both short- and longer-term gains.

Uganda commissioned its first National Community Health Strategy (NCHS) in March 2023 whose strategic directions include (i) Digitalizing CHW's reporting, (ii) Equipping CHWs with work tools, commodities and supplies, (iii) Strengthening supportive supervision, (iv) Compensating CHWs appropriately for work on a monthly or quarterly basis as will be determined operationally (21). The journey begun with the Community Health Roadmap that was launched by the Director General of Health Services (Dr. Henry Mwebesa) in June 2019, including among six priorities, to motivate and incentivize/ compensate CHWs, and aligning to global health development as the PHC rebirth in Astana (2018).

Relatedly in 2018, a Community Health Extension Worker (CHEW) strategy and policy were developed, and a pilot commenced in two districts and one city in 2022. The CHEW is a supervisory cadre of the community health workforce who will supervise VHTs and any other health-related village volunteers to strengthen their activities of reporting, health education, health promotion, and related mandates. Typically, two CHEWs are deployed at a parish, each supervising between six to twelve VHTs, and meriting the role after being selected by their community and vetted by district leaders using the criteria where candidates must hold a Senior Four (Ordinary Level) certification guaranteeing English literacy and living in the local community where they are willing to work. CHEWs are compensated with a monthly stipend worth UGX150,000/= monthly. The efforts suggest a government's commitment to harnessing evidence for compensating CHWs.

Despite this clear policy direction, limited empirical evidence exists on how different CHW compensation mixes influence performance and motivation within government-aligned systems. To address this gap, Living Goods Uganda implemented a **five-arm quasi-experimental study** to test varying pay structures—70:30, 50:50, and 30:70 performance-based incentive (PBI) to stipend ratios, plus a 100% stipend arm. The study assessed which mix best balances performance, motivation, and retention while ensuring sustainability and policy relevance. Findings from this experiment provide timely evidence to guide Uganda's National Community Health Strategy and inform similar initiatives in other low- and middle-income countries seeking effective, equitable CHW incentive models.

## 2 Context (setting and population in which the innovation occurs)

The World Health Organization's 2018 guidelines on Community Health Workers (CHWs) emphasize that effective national CHW programs must include a mix of financial and non-financial incentives, coupled with strong supervision and supportive systems. Uganda's policy trajectory has aligned closely with this global guidance, with an increasing commitment to compensate CHWs while addressing persistent challenges of volunteerism, weak supervision, and high attrition.

In March 2023, Uganda launched its first National Community Health Strategy (NCHS, 2020/21–2024/25), which outlined four strategic directions: (i) digitalizing CHW reporting systems; (ii) equipping CHWs with work tools, commodities, and supplies; (iii) strengthening supportive supervision; and (iv) compensating CHWs appropriately for their work, either monthly or quarterly. The strategy marked a turning point from a fully voluntary model to one that acknowledged the value of structured financial incentives as part of national community health system strengthening.

Earlier, in 2018, the Ministry of Health developed a Community Health Extension Worker (CHEW) policy to introduce a professionalized supervisory cadre. The CHEW pilot was implemented in 2022 across two districts and one city. Each parish-level CHEW supervises between six to twelve Village Health Teams (VHTs). Candidates are required to have a minimum of Senior Four (Ordinary Level) education to ensure English literacy and must reside in the community they serve. Importantly, CHEWs are compensated with a stipend of UGX 150,000 per month (≈ USD 40), signaling the government's readiness to formalize compensation for frontline health workers. This initiative reflects both the policy appetite and the political will to institutionalize CHW financing within Uganda's primary health care system.

At the same time, Living Goods, an NGO working in Uganda and Kenya, had been experimenting with financial compensation models since 2018. Its CHWs were remunerated through performance-based incentives (PBIs) tied to specific health outputs, such as household visits, treatment of sick children, follow-up of immunization defaulters, and family planning uptake. However, during the COVID-19 pandemic, Living Goods adapted its approach: the unpredictability of field conditions and increased workloads led to a shift from pure performance-based pay to blended models, combining fixed stipends with PBIs. This transition not only cushioned CHWs during service delivery disruptions but also provided valuable lessons on balancing income stability with incentivizing performance.

These overlapping policy and programmatic contexts—Uganda's NCHS and CHEW pilot, alongside Living Goods' evolving blended compensation system—created fertile ground for testing different financial incentive models in this quasi-experiment. The five districts selected represented a cross-section of rural Uganda, where VHTs are the first point of contact for families. The experiment was thus embedded in a real policy transition moment, with direct relevance for the government's long-term vision to incorporate CHW compensation into national health financing structures.

## 3 Detail to understand key programmatic elements

The quasi-experiment was conducted over a period of nine months across five Living Goods operational districts in Uganda. Each district was randomly allocated to one of five compensation arms designed to test different balances of fixed stipends and performance-based incentives (PBIs). The aim was to assess how varying incentive structures influenced Community Health Worker (CHW) performance, motivation, and retention, and to generate lessons for Uganda's emerging national CHW compensation policy framework.

### 3.1 Orientation and capacity building

Before the rollout, CHWs, peer supervisors, and site leaders underwent a structured one-month orientation tailored to their assigned compensation model. This training included modules on key performance indicators (KPIs), data reporting using smartphones and District Health Information System, Version 2 (DHIS2)-linked applications, supervision protocols, and clarification of performance thresholds. The orientation period was critical, as findings indicated that CHWs required an average of four to five months to fully understand and adapt to performance-based incentive systems. This highlighted the importance of structured onboarding and adaptation periods for future scale-up.This preparatory phase was critical. Findings from implementation showed that CHWs required an average of four to five months to fully adapt to performance-based models, underscoring the importance of structured onboarding and lag periods in future scale-up.

### 3.2 Compensation arms and modalities

The five compensation arms are summarized in Table 1. The specific pay-mix ratios were designed to reflect realistic operational scenarios and to test how different balances of fixed stipends and performance-based incentives (PBIs) influence CHW motivation, performance, and retention. The **50:50 arm** represented Living Goods' standard operational model and therefore served as the *control*. The **70% PBI arm** tested an intensive, performance-oriented design emphasizing accountability for key outputs. The **30% PBI arm** represented a lower-intensity incentive structure, focusing on income stability and intrinsic motivation. The **100% stipend arm** modeled a government-feasible fixed-income comparator without performance linkage. All arms were standardized to an equivalent total value of approximately **US $ 20 per month (UGX ≈ 70,000)** to ensure cost realism and comparability across districts.

**Table 1:** Compensation arms, modalities, and monthly stipend amounts by site.

Arm	Site	Fixed Stipend Component	PBI Component	Description
Arm 1	Kira	50%	50%	Balanced model.
Arm 2	Wobulenzi	70%	30%	High-stipend, low-PBI arm testing income predictability.
Arm 3	Masajja	50%	50%	Replication arm for control condition.
Arm 4	Budadiri	30%	70%	High-performance arm testing stronger PBI weighting.
Arm 5	Mpigi	100%	0%	Pure stipend comparator for government feasibility.

### 3.3 CHW allocation and sample distribution by site

Each compensation arm was piloted in a different site, with baseline and endline assessments conducted to provide robust longitudinal data on participation and performance. In Kira, the control arm tested a balanced model of 50% fixed stipend and 50% performance-based incentives (PBIs), enrolling 134 CHWs at baseline and 135 at endline. Budadiri piloted the high-intensity PBI model in which 70% of income depended on key performance indicators (KPIs) and 30% was a fixed stipend, involving 147 CHWs at baseline and 150 at endline. Masajja implemented an equal split model (50% stipend, 50% KPIs), with 136 CHWs at baseline and 151 at endline. In Mpigi, the stipend-only arm offered a 100% fixed stipend with no performance-based component, enrolling 146 CHWs at baseline and 147 at endline. Finally, Wobulenzi tested the predominantly stipend-based model with 70% fixed stipend and 30% KPIs, engaging 126 CHWs at baseline and 149 at endline. Together, these distributions ensured that the study captured the effects of different compensation structures across varied geographical and operational contexts.

### 3.4 Supervision and support structures

Each arm was supported with a multi-layered supervision framework to ensure fidelity and accountability. This included:

- Monthly supervision visits by Living Goods program officers;
- Bi-weekly peer supervision at the community level; and
- Weekly peer-group meetings in which clusters of six to ten CHWs shared experiences, monitored progress, and received refresher coaching.

Peer supervisors were themselves incentivized, with part of their income linked to the performance of the CHWs they supervised. This design strengthened accountability while reinforcing mentorship and performance monitoring across all sites.

### 3.5 Data collection and monitoring

The study employed a five-arm quasi-experimental design with mixed quantitative and qualitative methods. Baseline surveys covered 689 CHWs and 2,315 households, while endline surveys assessed 732 CHWs and 2,339 households. In addition, 62 key informant interviews were conducted with Ministry of Health officials, district health officers, local leaders, and program managers, alongside 11 focus group discussions with CHWs and community members. Programmatic data from Living Goods' digital tools and the national DHIS2 system were analyzed to track service delivery indicators such as household visits, treatment of sick children, family planning referrals, immunization defaulter follow-up, and postnatal care checks.

The study pre-specified three primary outcomes: CHW performance, defined as achievement of at least five of eight monthly key performance indicators (KPIs); CHW motivation, measured using the validated *Close-to-Community Provider Motivation Scale* (22); and CHW retention, defined as continuous active reporting throughout the nine-month intervention period. Secondary outcomes included specific service coverage metrics such as household visitation, under-five assessments, defaulter tracking, family-planning referrals, and postnatal checks.

The total sample comprised 1,104 CHWs distributed across five study arms. The distribution of CHWs across the five compensation arms at baseline and endline is presented in Table 2. Power calculations (*α* = 0.05; 80% power) were based on detecting a 10-percentage-point difference in performance between arms, assuming an intracluster correlation coefficient (ICC) of 0.05 and cluster sizes of 130–150 CHWs per site. This provided sufficient power to detect an effect size of roughly 0.25 standard deviations for continuous outcomes.

**Table 2:** CHWs' compensation structure and CHWs assessed at baseline and endline.

Compensation Arm/ Structure	Site Name	Baseline (N1)	Endline (N2)
50% Stipend, 50% Activity (Control Arm)	Kira	134	135
30% Stipend, 70% KPIs	Budadiri	147	150
50% Stipend, 50% KPIs	Masajja	136	151
100% Stipend, No KPIs	Mpigi	146	147
70% Stipend, 30% KPIs	Wobulenzi	126	149

**Figure 1:**
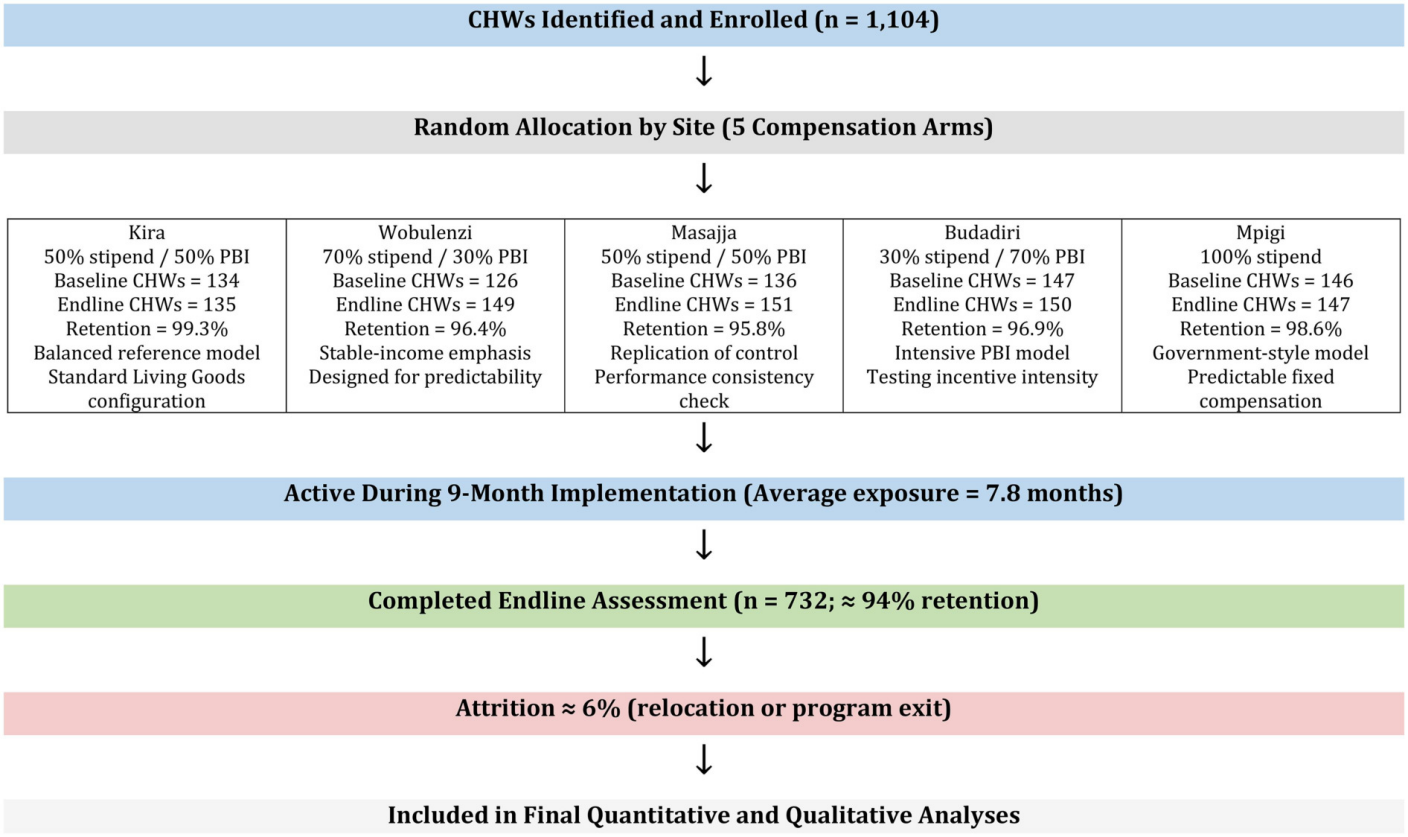
Participant flow diagram for the quasi-experiment.

A simplified CONSORT-style flow diagram (Figure 1) illustrates participant progression from enrolment through baseline, implementation, and endline assessment. Attrition was low (∼ 6%), mainly due to relocation or routine program exits. These clarifications improve transparency on study design, sampling, and pre-specified outcomes, ensuring methodological rigor and reproducibility.

A total of 689 CHWs participated in baseline assessments and 732 CHWs were assessed at endline. The slight increase resulted from mid-term replacements of inactive or relocated CHWs, maintaining full operational coverage across arms. Overall attrition was approximately 6%, primarily due to relocation, family obligations, or transition to other employment.

On average, CHWs were exposed to their assigned compensation model for 7.8 months, with minimal variation between study sites. Performance tracking data showed that most improvements in service coverage occurred after the third month of implementation, corresponding to the period when CHWs had fully adapted to performance-linked pay systems.

A dose–response pattern was observed between months of active engagement and performance scores: CHWs with longer uninterrupted participation achieved higher KPI attainment and motivation ratings. Supplementary Appendix A provides descriptive plots illustrating this relationship, highlighting how consistent exposure to the compensation model was associated with incremental improvements in CHW productivity and reporting compliance. These findings emphasize the importance of continuous engagement and adaptation time in performance-based compensation schemes. Table 3 summarizes the difference-in-difference estimates across key performance indicators for the five compensation arms.

**Table 3:** Performance summary (difference in difference- coefficients).

Indicator	70%KPI	50%KPI	Stipend	30%KPI
LG Program data (38months)	Adj.Coef, *p*-value	Adj.Coef, *p*-value	Adj.Coef, *p*-value	Adj.Coef, *p*-value
% sick child referrals	−0.038, 0.008**		−0.105, 0.002**	
% unique HH visits	0.030, 0.458	−0.031, 0.046*	0.006, 0.886	0.030, 0.458
FP coverage (%)	0.066, 0.008**		−0.128, 0.033*	0.063, 0.010*
# FP referrals f/up	1.096, 0.007**			
Household data (2,315, 2,339)				
% HH with sick under-five ≤2 weeks	−0.189, 0.000**	0.143, 0.000**	0.171, 0.000**	
% up-to-date vaccination ≤23 mo	−0.080, 0.044*	0.084, 0.032*		−0.122, 0.004**

*p < 0.05, **p < 0.01.

### 3.6 Data analysis

We applied a difference-in-differences (DiD) model to estimate the intervention effects on key outcomes. The model specification was:

$$Y_{it}=\beta_{0}+\beta_{1}Post_{t}+\beta_{2}Treatment_{i}+\beta_{3}(Post_{t}\times Treatment_{i})+X_{it}\gamma +\varepsilon_{it}$$

where $Y_{it}$ represents each CHW's outcome at time *t*, and $X_{it}$ includes key covariates.

Standard errors were clustered at the district level (*n* = 5), and statistical inference used wild-cluster bootstrap *p*-values for small-cluster correction. We report 95% confidence intervals and apply Benjamini–Hochberg adjustments for multiple testing.

Pre-trend graphs using monthly program data were added to confirm parallel trend assumptions (Supplementary Appendix B, Supplementary Figures A1–A3). These figures demonstrate that pre-intervention slopes across arms were broadly similar, supporting validity of the DiD design.

All analyses were conducted in R and Stata, with sensitivity checks using unadjusted and covariate-adjusted models yielding consistent results.

- DID model in the table is based on the Intention-to-Treat (ITT) model
- The alternative, As-treated model (basing on September 2023 as start of treatment/ intervention) has fewer performance results.

**Figure 2:**
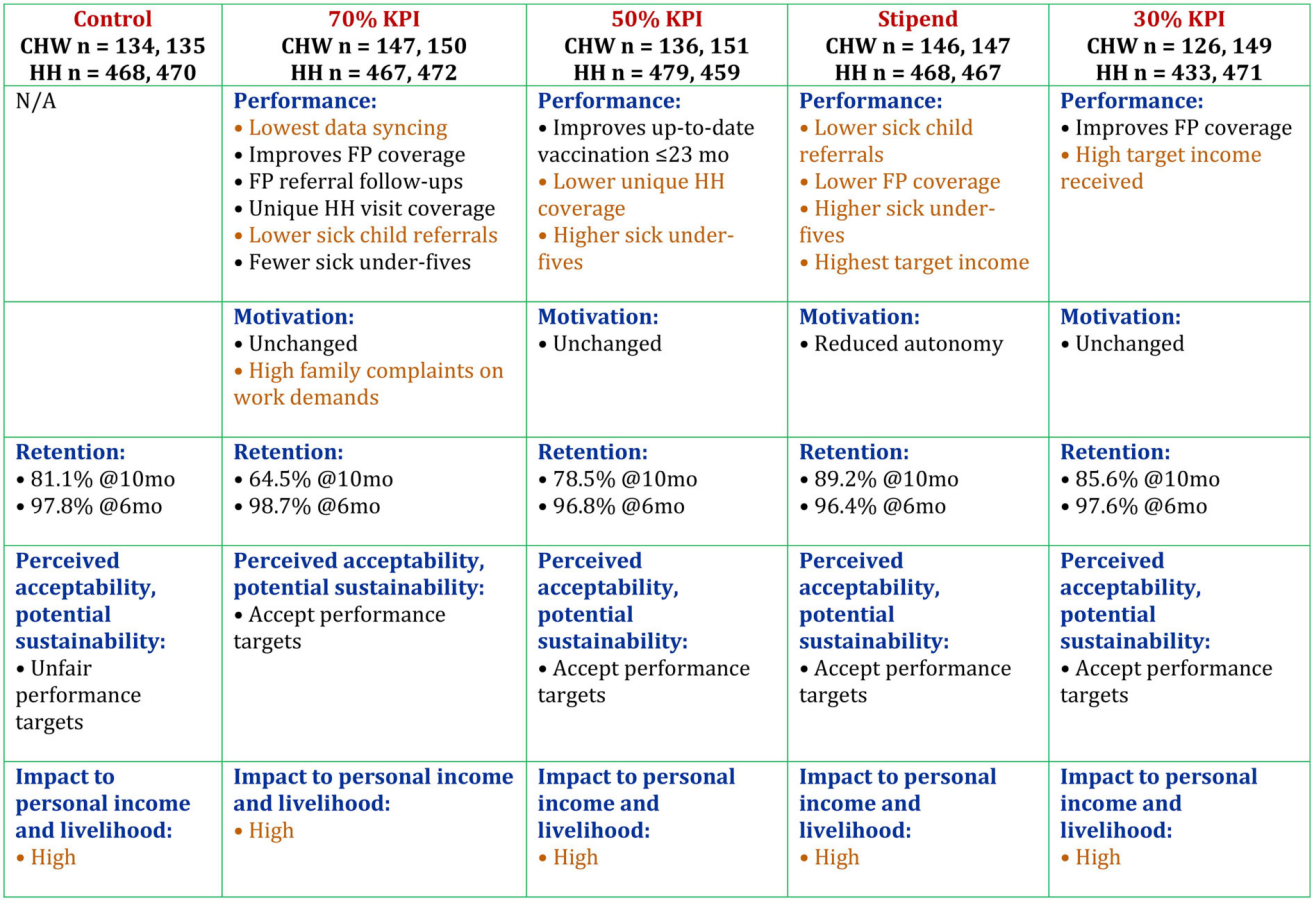
Overall summary of the results.

Figure 2 presents a consolidated summary of the key study outcomes across the five compensation arms. It visually compares performance indicators (such as sick-child referrals, household visitation and family-planning follow-up), CHW motivation scores, six- and ten-month retention levels, perceived acceptability and sustainability of performance targets, and the perceived impact of each compensation model on personal income. This figure synthesizes the main quantitative and qualitative findings for each arm, making cross-arm differences easy to interpret.

The Intention-to-Treat difference-in-difference estimates for the key performance indicators are presented in Table 3. This table compares the four compensation arms with the control group, showing how each model influenced CHW performance outcomes using program data and household survey indicators. Positive adjusted coefficients indicate improvement relative to control over the eight-month intervention period, while negative coefficients indicate a relative decline. Statistically significant effects are highlighted by *p*-values below conventional thresholds. Overall, Table 3 summarizes how the 70% KPI, 50% KPI, stipend, and 30% KPI arms differentially affected sick-child referrals, household visitation coverage, family-planning follow-up, and up-to-date vaccination among children under 23 months.

### 3.7 Challenges and implementation fidelity

Several challenges were observed during implementation. There were differences in baseline performance across districts, which affected comparability between arms. In addition, CHWs required a longer adaptation period before they could fully internalize the logic of PBIs, creating delays in programmatic fidelity. Variation in the consistency of supervision visits, periodic stock-outs of essential commodities, and intermittent digital reporting challenges were also noted across sites, occasionally affecting data flow and CHW performance monitoring. The complexity of monitoring PBIs also introduced risks of data falsification and reporting inaccuracies, highlighting the importance of strong verification mechanisms in future scale-up. Despite these challenges, the compensation models were rolled out as planned, generating valuable insights into how CHW performance, motivation, and retention respond to different mixes of fixed stipends and PBIs.

## 4 Discussion: practical implications and lessons learned for future applications

This quasi-experiment provides a rare opportunity to operationally assess how different CHW compensation structures influence performance, motivation, and retention. Unlike theoretical frameworks or small-scale studies, this pilot was embedded within a large-scale program, implemented over nine months with sufficient rigor to draw policy-relevant lessons. The findings directly inform Uganda's National Community Health Strategy (2020/21–2024/25), which calls for regular CHW compensation but leaves open the question of structure—whether through stipends, performance-based payments, or a hybrid model.

### 4.1 Practical implications

The study confirms that no single model provides a perfect solution. The stipend-only approach offered predictability and the highest consistency in income targets and data syncing, which CHWs valued as reliable support for household stability. However, this model consistently underperformed in key service delivery areas, including family planning (FP) coverage, sick child assessments, and postnatal care. Without a performance linkage, CHWs appeared less motivated to sustain outreach intensity, reducing service delivery effectiveness.

By contrast, the high-threshold performance model, where 70% of income was tied to KPIs, produced the strongest improvements across FP coverage, household visits, referral follow-ups, and under-five illness assessments. Household coverage exceeded 85% of targeted homes during the intervention period. Yet these gains came at a cost: many CHWs reported stress, domestic strain, and burnout. Retention also dropped after the tenth month, suggesting that sustained pressure without psychosocial support risks attrition.

Motivation dynamics were central to these outcomes. While performance-based pay improved measurable outputs such as household visits and child assessments, it also shifted CHWs' focus toward meeting numeric targets rather than providing holistic care. In the hybrid arms (30%–50% PBI), CHWs reported feeling both recognized and secure, combining the reassurance of a fixed stipend with the motivation of achievable performance rewards. Conversely, CHWs in high-PBI arms described anxiety, perceived competition, and family strain, which eroded teamwork and confidence over time. These dynamics illustrate how moderate incentive structures can sustain engagement without undermining intrinsic motivation, echoing the mixed results observed in the quantitative motivation scores and qualitative feedback from CHWs and supervisors.

The 50% stipend/50% KPI model produced moderate success, particularly in child immunization, but did not match the stronger results of the 70% model. Meanwhile, the 30% KPI arm underperformed and even showed declines in outcomes such as facility deliveries and timely child vaccinations, indicating that too small a performance-based component may fail to sustain accountability.

Taken together, these findings suggest that hybrid models with moderate PBIs (30%–50%) are most effective for balancing predictability, motivation, and performance. Retention across most arms remained high at six months (95%–97%), demonstrating that stipends anchor CHW engagement. However, motivation and retention deteriorated in high-performance-heavy arms over time, reinforcing the need for a balanced approach.

From a policy perspective, three broad options emerge. A stipend-only model is administratively straightforward and financially predictable but risks lowering outputs. A performance-only model maximizes accountability but creates risks of stress, fraud, and attrition. A hybrid model—combining stipends with moderate PBIs—offers the most balanced pathway. This aligns with Uganda's policy goals but requires robust monitoring, fraud prevention, and integration with digital platforms such as DHIS2 and Electronic Community Health Information System (eCHIS).

### 4.2 Lessons learned

Several lessons stand out from the implementation. First, adaptation to performance-based models takes time. CHWs and supervisors required four to five months to fully internalize the system, underscoring the need for dedicated lag periods for training, coaching, and adaptation in any future rollout. Without this, new schemes risk early disengagement and weak fidelity.

Second, supervision structures proved pivotal. The layered approach of monthly program officer visits, bi-weekly peer supervision, and weekly peer-group meetings created accountability, reduced under-performance, and promoted peer learning. Incentivizing peer supervisors further strengthened this model, offering a replicable framework for national policy.

Third, motivation was shaped by more than income. CHWs highlighted the importance of supportive supervision, provision of medicines and smartphones, branded uniforms, and a sense of recognition. One CHW explained: “*It*”*s not just about the money. When they check on us and talk to us nicely, it makes us feel like we’re part of something important—not just people who are given money to do things”*. Such non-financial enablers reinforced intrinsic motivation and should remain integral to CHW programs.

Fourth, autonomy emerged as essential for job satisfaction. CHWs in performance-based arms valued the sense of ownership that targets provided, while some in the stipend-only arm felt reduced independence, likening the system to a government salary structure. Policies must therefore balance financial security with respect for CHW autonomy and ownership.

Fifth, sustainability was a recurrent concern among stakeholders. While performance incentives were valued for accountability, government stakeholders questioned whether high-resource models such as Living Goods' could be replicated without donor support. Risks of data falsification under high-PBI models were also noted, underscoring the need for strong fraud monitoring, realistic target-setting, and investment in digital verification.

Finally, retention patterns revealed important risks. Although retention was strong at six months, attrition increased notably at ten months under high-performance arms. This suggests that performance-based models must integrate wellness strategies, including stress management support, realistic targets, and continuous coaching, to prevent burnout and sustain CHW engagement.

Gender, ethics, and sustainability considerations. The majority of CHWs in this quasi-experiment were women balancing caregiving and community health duties, making compensation models not only a financial issue but also one of gender equity and professional recognition. Predictable income through stipends reduced financial stress and supported household stability, while performance-linked pay fostered a sense of purpose and achievement. However, excessive performance pressure risked ethical concerns such as data exaggeration and burnout. Sustaining motivation and fairness therefore requires hybrid models that embed both financial security and transparent performance monitoring. For long-term viability, integration of these models into government financing and digital verification systems—such as DHIS2 and eCHIS—will be critical to maintain accountability and equity as donor support declines.

### 4.3 Summary

Ultimately, the study demonstrates that CHW compensation cannot be approached as a one-size-fits-all model. A hybrid system combining stipends with moderate PBIs provides the most balanced outcomes, enhancing accountability while safeguarding motivation and retention. To succeed, such models must be embedded within strong supervision structures, adapted gradually with adequate onboarding periods, and supported by both financial and non-financial enablers. These findings provide timely evidence for Uganda's CHEW policy and National Community Health Strategy, while also offering practical lessons for other LMICs pursuing sustainable CHW compensation systems.

## 5 Acknowledgment of conceptual and methodological constraints

Several constraints must be acknowledged when interpreting the findings of this quasi-experiment. First, the adaptation period required for CHWs to fully understand and engage with the performance-based incentive structures was longer than anticipated. Most CHWs required four to five months to internalize the system, creating a lag that reduced programmatic fidelity in the early stages. This has implications for the interpretation of short-term results and highlights the need for extended onboarding in future rollouts.

Second, the study was conducted over a relatively short implementation window of nine months. While this duration was sufficient to observe initial impacts on performance, motivation, and retention, it limited the ability to assess long-term outcomes such as sustained retention, cumulative stress effects, and the durability of performance gains.

Third, differences in baseline performance levels across sites complicated comparability between arms. While random allocation of arms across districts was designed to minimize bias, contextual variations in CHW capacity, community demand, and supervisory environments may have influenced outcomes.

Fourth, the complexity of monitoring performance-based incentives introduced risks of data falsification and reporting inaccuracies. These risks were particularly noted in arms with high proportions of performance-linked pay, underscoring the importance of strong verification systems in interpreting results.

Fifth, the Living Goods model is resource-rich, supported by intensive supervision, digital reporting, and donor funding. Stakeholders noted that while the findings are valuable, replicating such a model under government financing may pose challenges. As such, questions remain about the scalability and affordability of certain elements, particularly in resource-constrained settings.

Finally, while the results are highly relevant for Uganda's policy context, generalizability to other low- and middle-income countries must be approached with caution. Differences in health system structures, financing frameworks, and CHW program designs may limit direct transferability of these findings without local adaptation.

Taken together, these constraints do not diminish the value of the study but rather frame its lessons within the realities of implementation research. They highlight the importance of cautious interpretation, contextual adaptation, and further longitudinal evaluation to build a more complete evidence base for CHW compensation models.

## Data Availability

The raw data supporting the conclusions of this article will be made available by the authors, without undue reservation.
