# Supplementary material for: Effects of financial compensation structures on community health worker performance, motivation, and retention: evidence from a multi-arm quasi-experiment in Uganda
Source: Front Health Serv. 2025 Dec 11;5:1687782. doi: 10.3389/frhs.2025.1687782 (PMC12738377; doi:10.3389/frhs.2025.1687782)
Supplement: Supplementary file 1 [file Presentation1.pptx]

## Slide 1
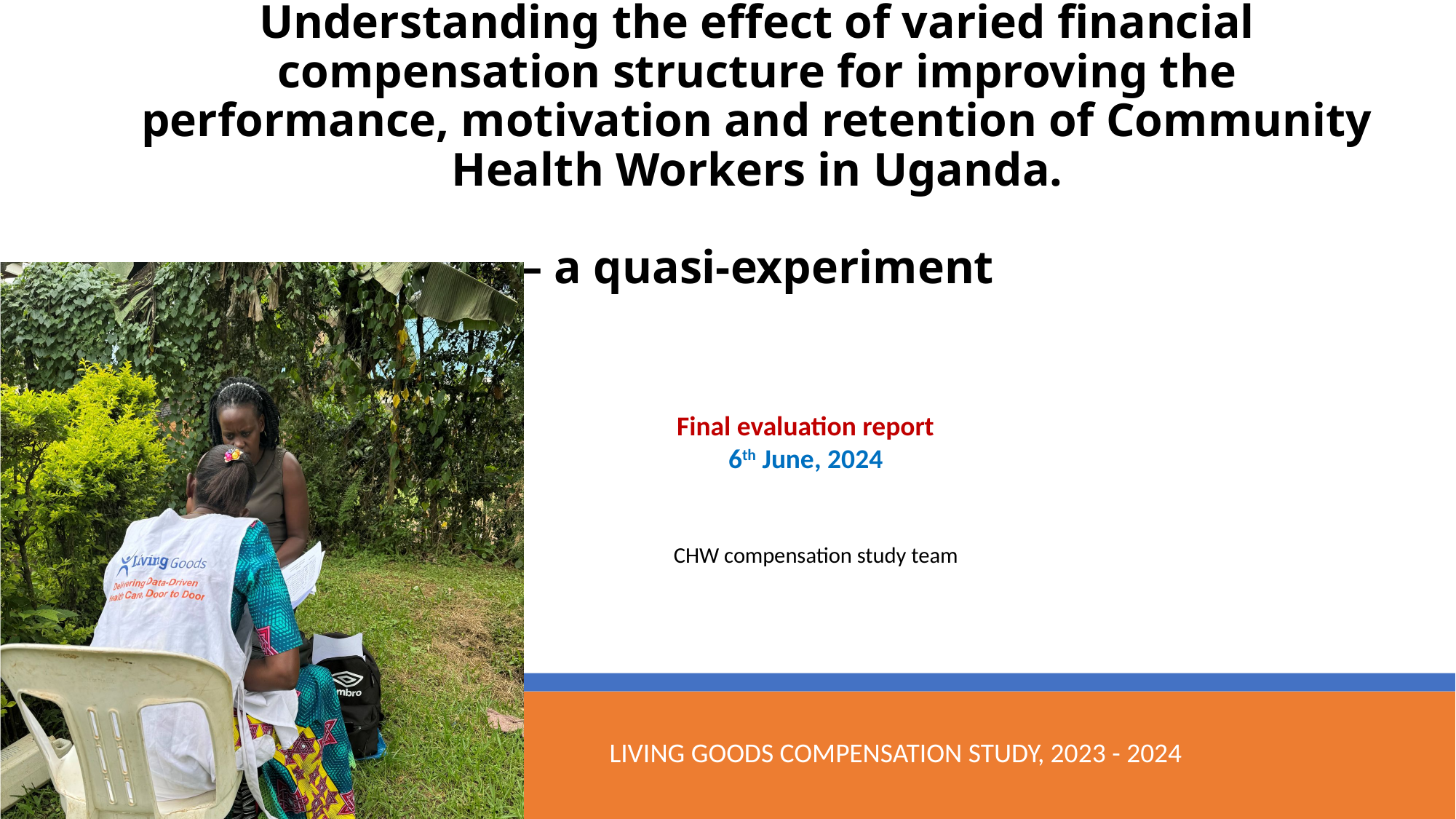

# Understanding the effect of varied financial compensation structure for improving the performance, motivation and retention of Community Health Workers in Uganda. – a quasi-experiment
Final evaluation report
6th June, 2024
CHW compensation study team
 Living goods compensation study, 2023 - 2024

## Slide 2
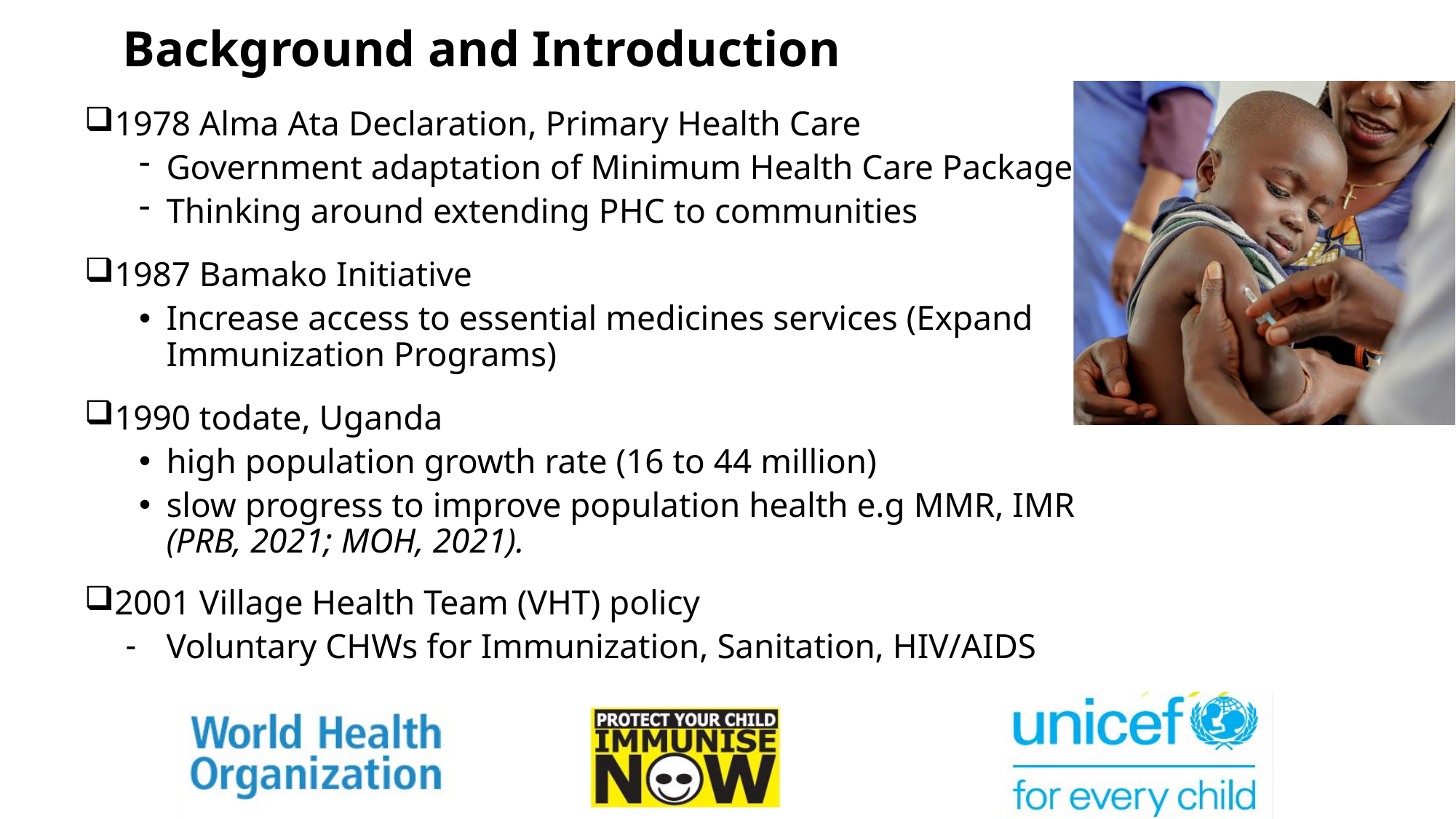

# Background and Introduction
1978 Alma Ata Declaration, Primary Health Care
Government adaptation of Minimum Health Care Package
Thinking around extending PHC to communities
1987 Bamako Initiative
Increase access to essential medicines services (Expand Immunization Programs)
1990 todate, Uganda
high population growth rate (16 to 44 million)
slow progress to improve population health e.g MMR, IMR (PRB, 2021; MOH, 2021).
2001 Village Health Team (VHT) policy
Voluntary CHWs for Immunization, Sanitation, HIV/AIDS

## Slide 3
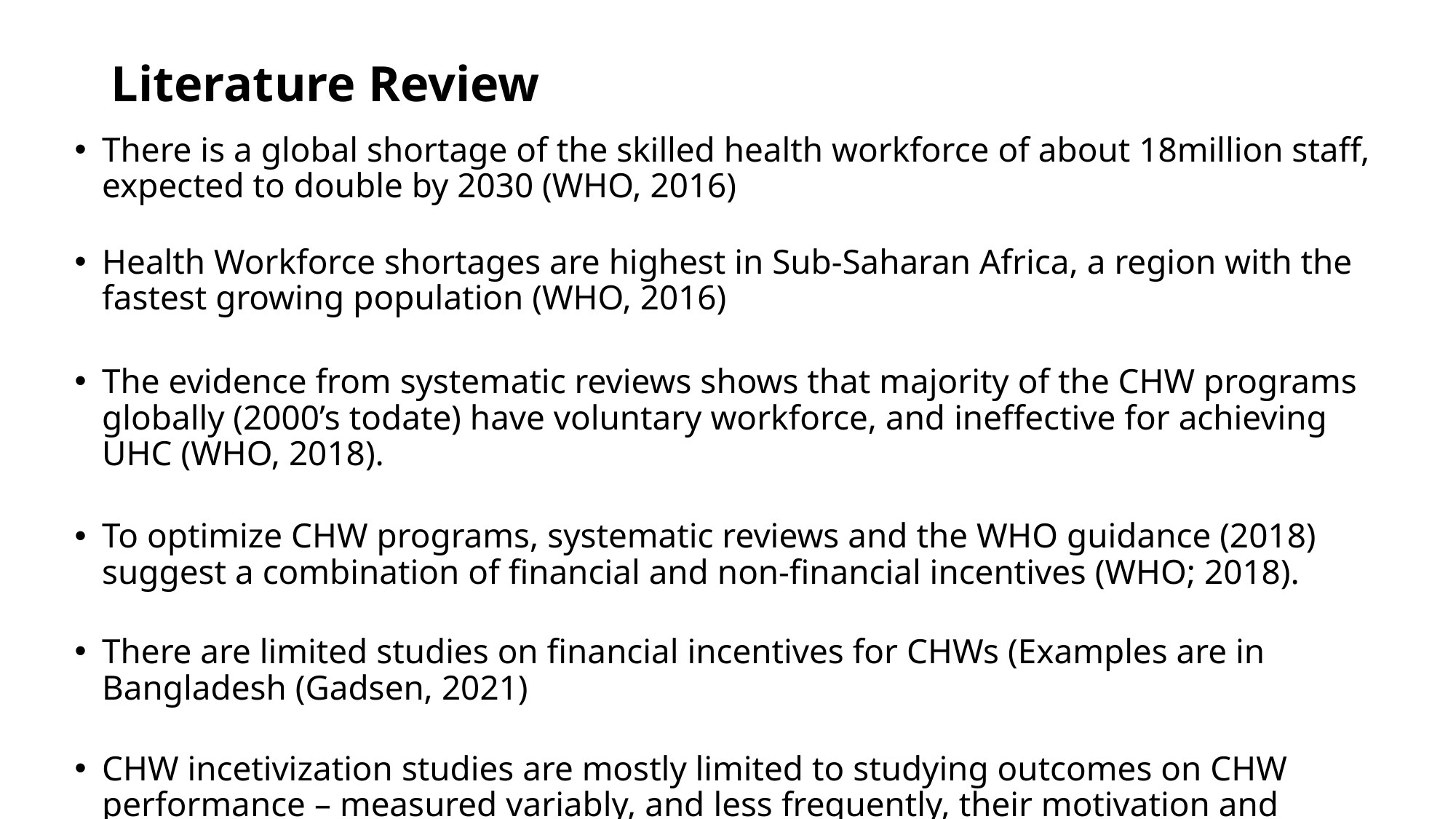

# Literature Review
There is a global shortage of the skilled health workforce of about 18million staff, expected to double by 2030 (WHO, 2016)
Health Workforce shortages are highest in Sub-Saharan Africa, a region with the fastest growing population (WHO, 2016)
The evidence from systematic reviews shows that majority of the CHW programs globally (2000’s todate) have voluntary workforce, and ineffective for achieving UHC (WHO, 2018).
To optimize CHW programs, systematic reviews and the WHO guidance (2018) suggest a combination of financial and non-financial incentives (WHO; 2018).
There are limited studies on financial incentives for CHWs (Examples are in Bangladesh (Gadsen, 2021)
CHW incetivization studies are mostly limited to studying outcomes on CHW performance – measured variably, and less frequently, their motivation and retention

## Slide 4
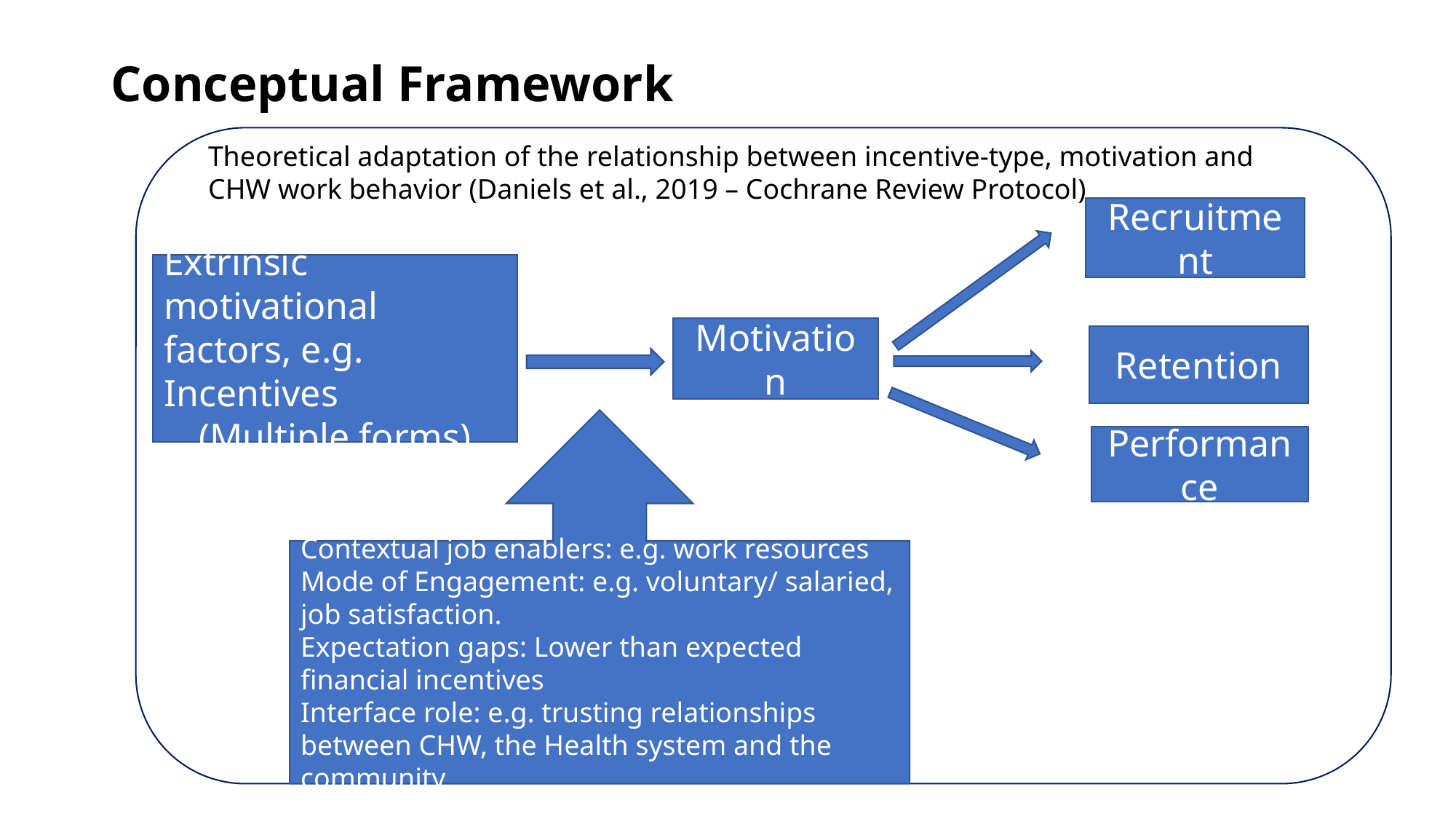

# Conceptual Framework
Recruitment
Extrinsic motivational factors, e.g. Incentives
(Multiple forms)
Motivation
Retention
Performance
Theoretical adaptation of the relationship between incentive-type, motivation and CHW work behavior (Daniels et al., 2019 – Cochrane Review Protocol)
Contextual job enablers: e.g. work resources
Mode of Engagement: e.g. voluntary/ salaried, job satisfaction.
Expectation gaps: Lower than expected financial incentives
Interface role: e.g. trusting relationships between CHW, the Health system and the community

## Slide 5
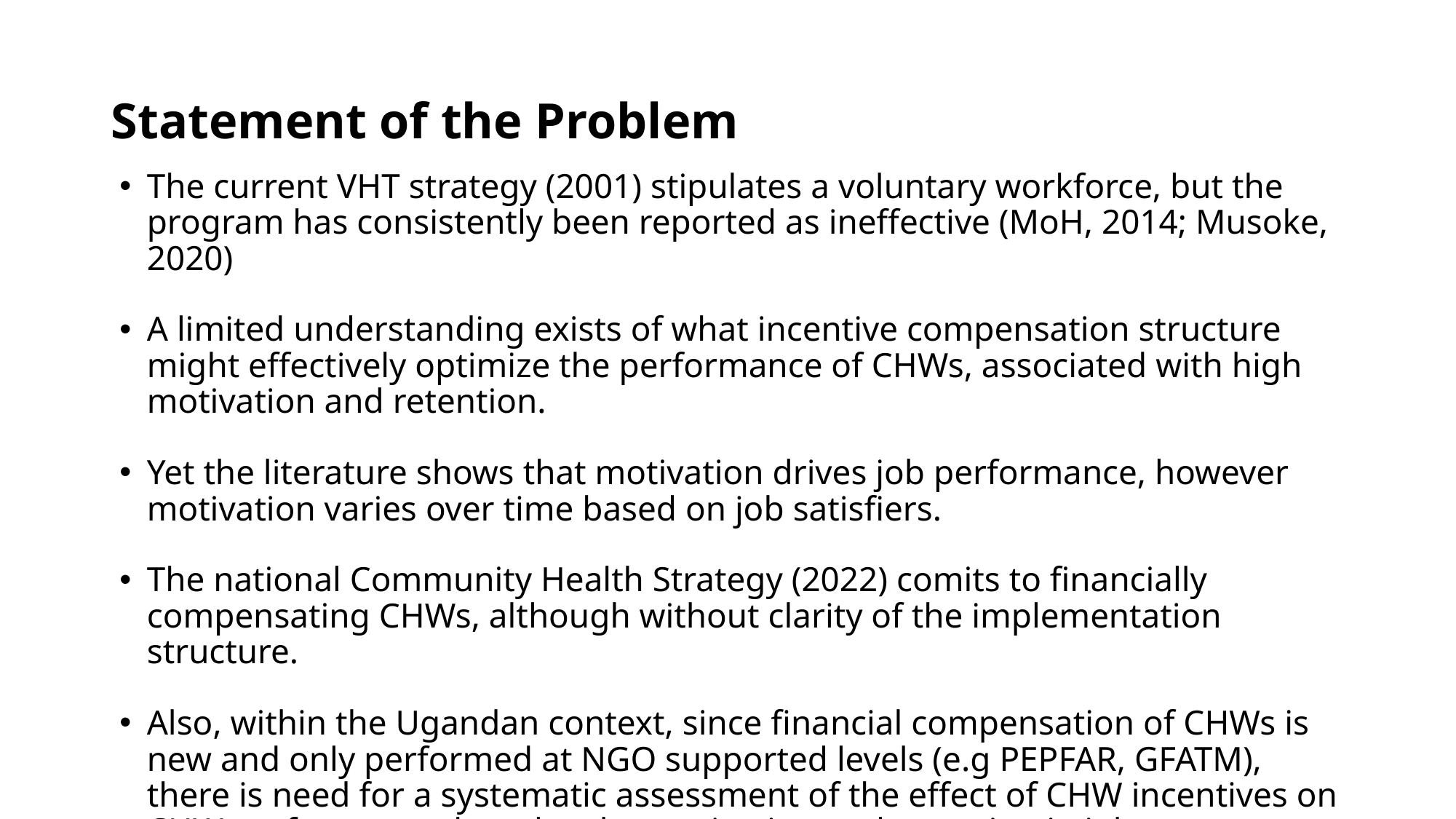

# Statement of the Problem
The current VHT strategy (2001) stipulates a voluntary workforce, but the program has consistently been reported as ineffective (MoH, 2014; Musoke, 2020)
A limited understanding exists of what incentive compensation structure might effectively optimize the performance of CHWs, associated with high motivation and retention.
Yet the literature shows that motivation drives job performance, however motivation varies over time based on job satisfiers.
The national Community Health Strategy (2022) comits to financially compensating CHWs, although without clarity of the implementation structure.
Also, within the Ugandan context, since financial compensation of CHWs is new and only performed at NGO supported levels (e.g PEPFAR, GFATM), there is need for a systematic assessment of the effect of CHW incentives on CHW performance, but also the motivation and retention in job.

## Slide 6
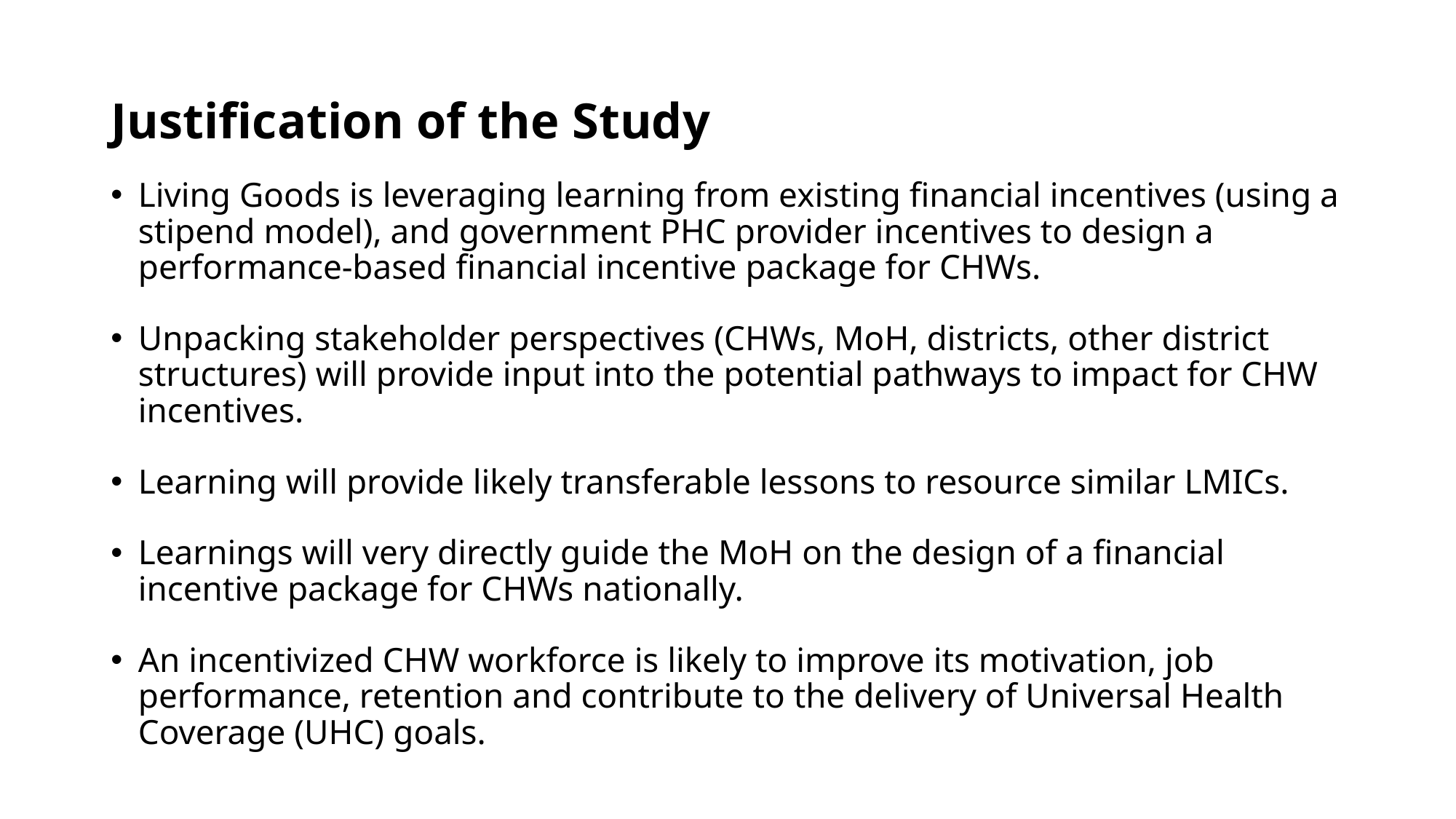

# Justification of the Study
Living Goods is leveraging learning from existing financial incentives (using a stipend model), and government PHC provider incentives to design a performance-based financial incentive package for CHWs.
Unpacking stakeholder perspectives (CHWs, MoH, districts, other district structures) will provide input into the potential pathways to impact for CHW incentives.
Learning will provide likely transferable lessons to resource similar LMICs.
Learnings will very directly guide the MoH on the design of a financial incentive package for CHWs nationally.
An incentivized CHW workforce is likely to improve its motivation, job performance, retention and contribute to the delivery of Universal Health Coverage (UHC) goals.

## Slide 7
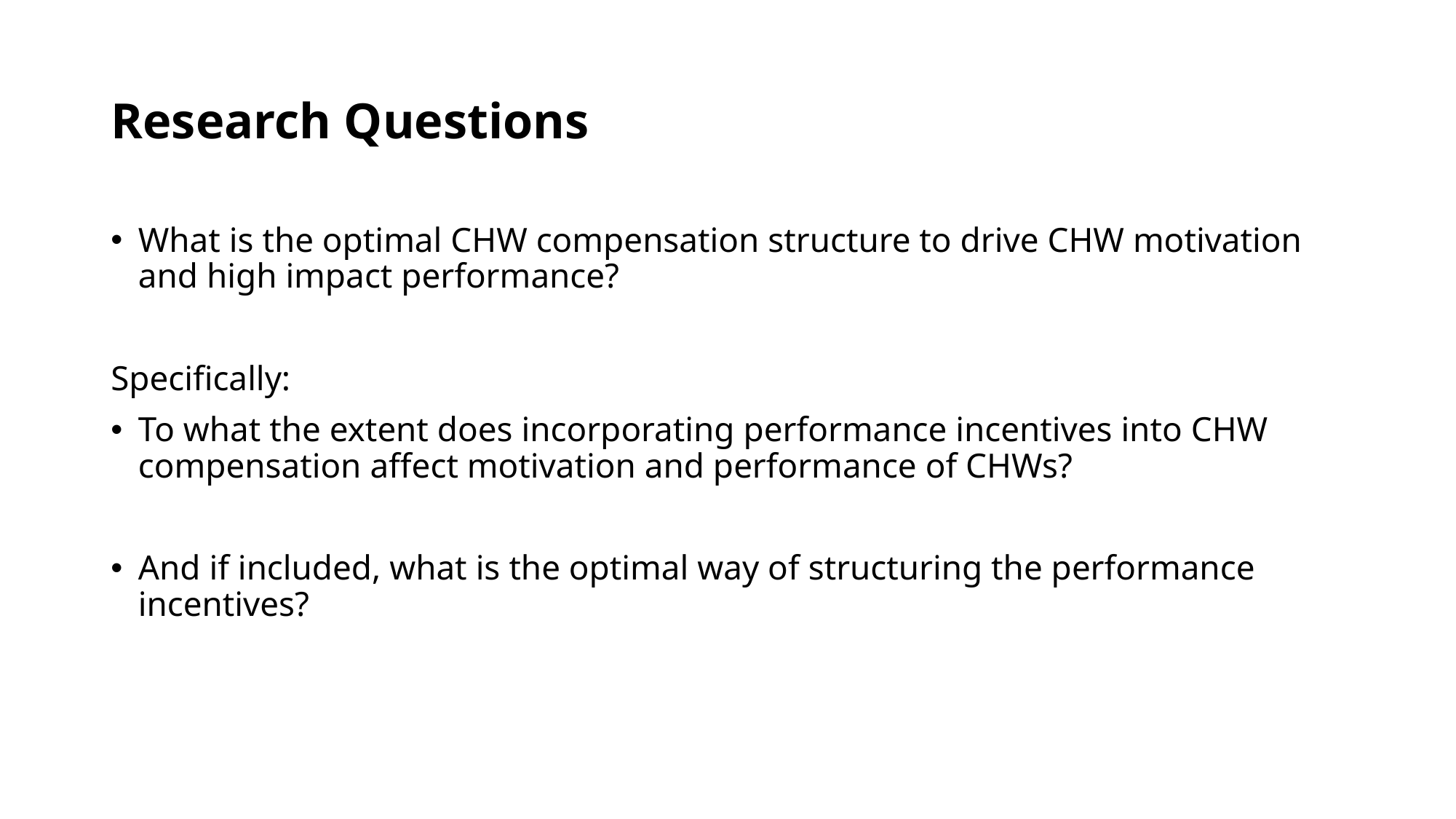

# Research Questions
What is the optimal CHW compensation structure to drive CHW motivation and high impact performance?
Specifically:
To what the extent does incorporating performance incentives into CHW compensation affect motivation and performance of CHWs?
And if included, what is the optimal way of structuring the performance incentives?

## Slide 8
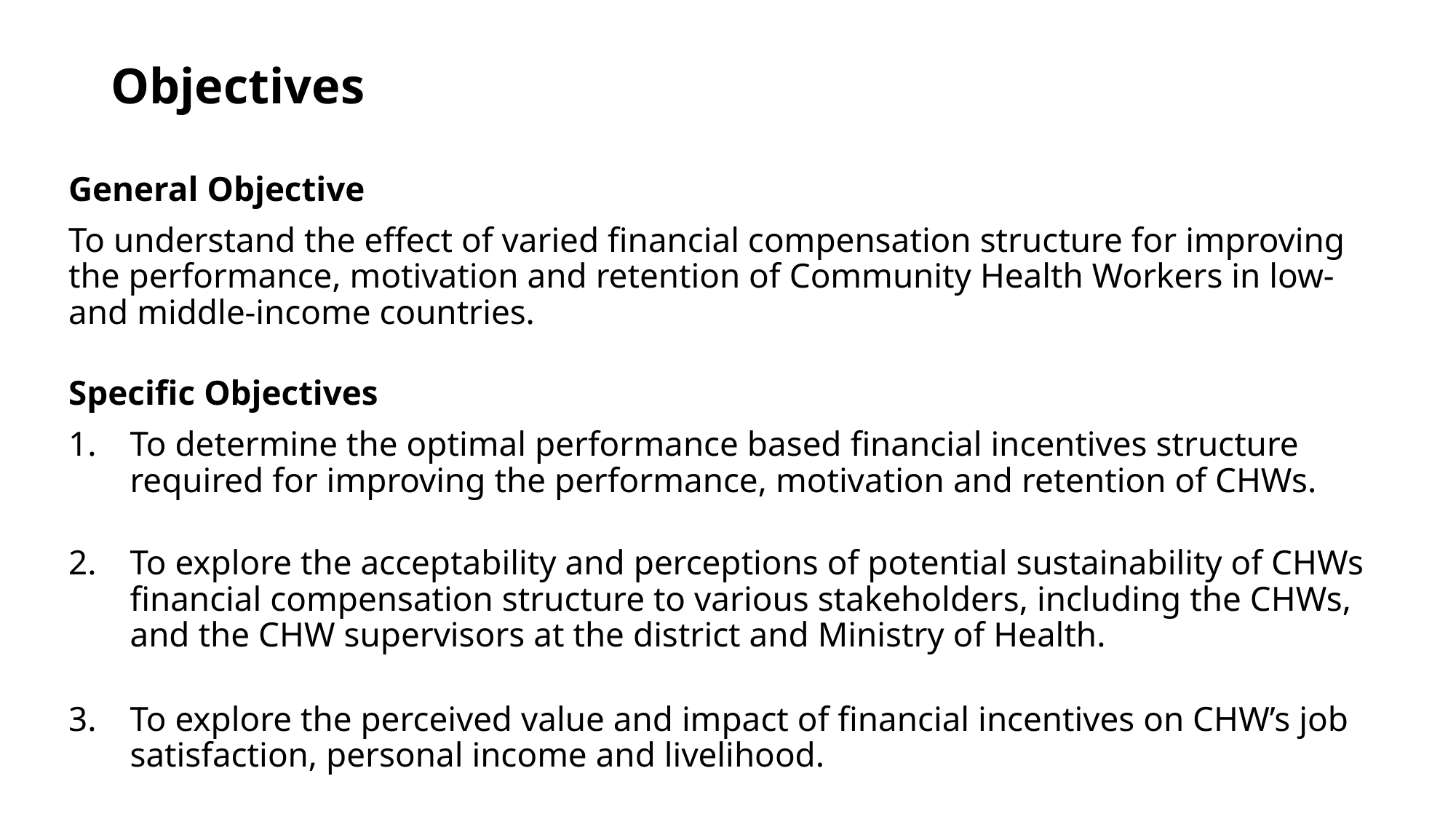

# Objectives
General Objective
To understand the effect of varied financial compensation structure for improving the performance, motivation and retention of Community Health Workers in low- and middle-income countries.
Specific Objectives
To determine the optimal performance based financial incentives structure required for improving the performance, motivation and retention of CHWs.
To explore the acceptability and perceptions of potential sustainability of CHWs financial compensation structure to various stakeholders, including the CHWs, and the CHW supervisors at the district and Ministry of Health.
To explore the perceived value and impact of financial incentives on CHW’s job satisfaction, personal income and livelihood.

## Slide 9
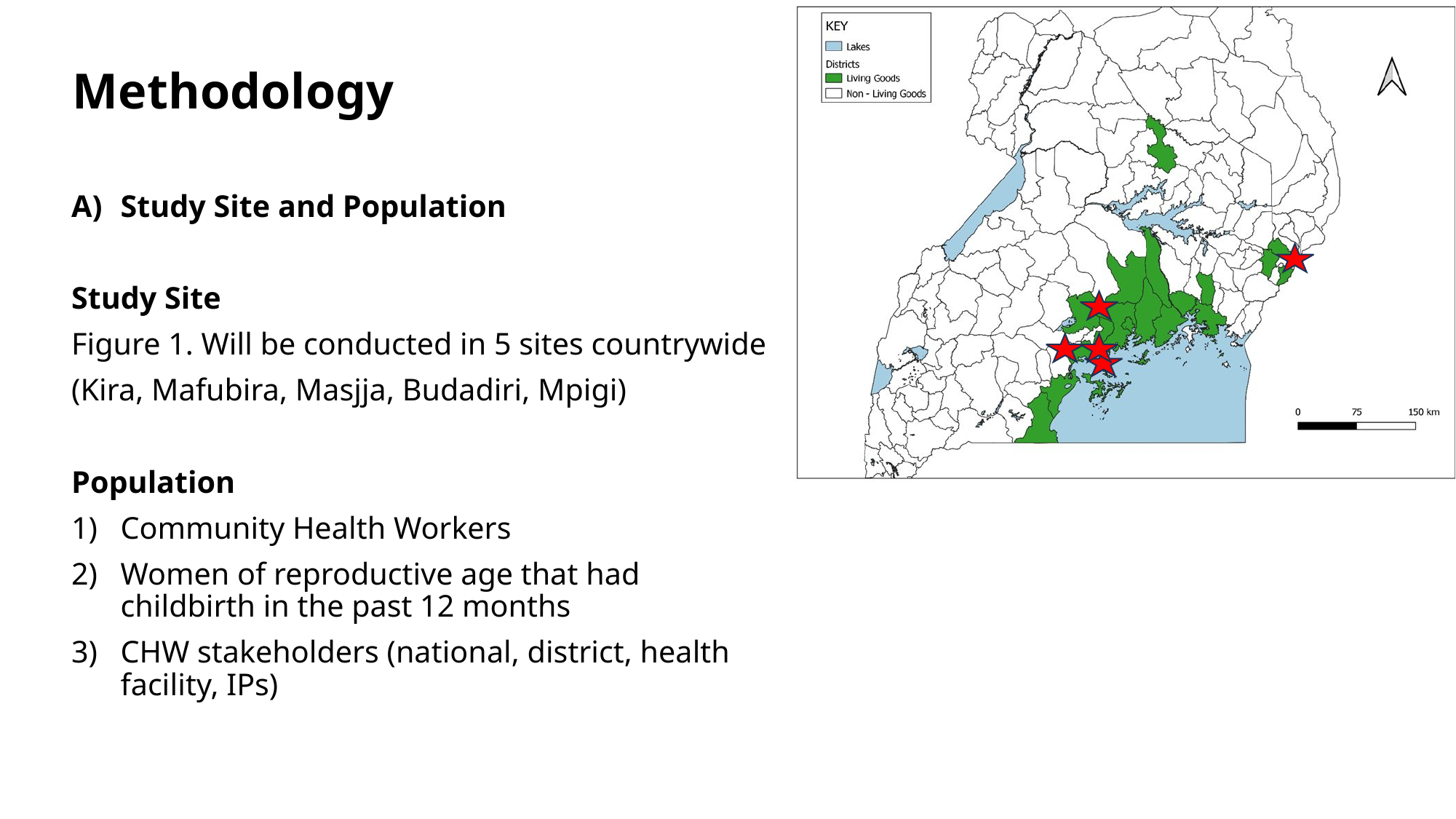

# Methodology
Study Site and Population
Study Site
Figure 1. Will be conducted in 5 sites countrywide
(Kira, Mafubira, Masjja, Budadiri, Mpigi)
Population
Community Health Workers
Women of reproductive age that had childbirth in the past 12 months
CHW stakeholders (national, district, health facility, IPs)

## Slide 10
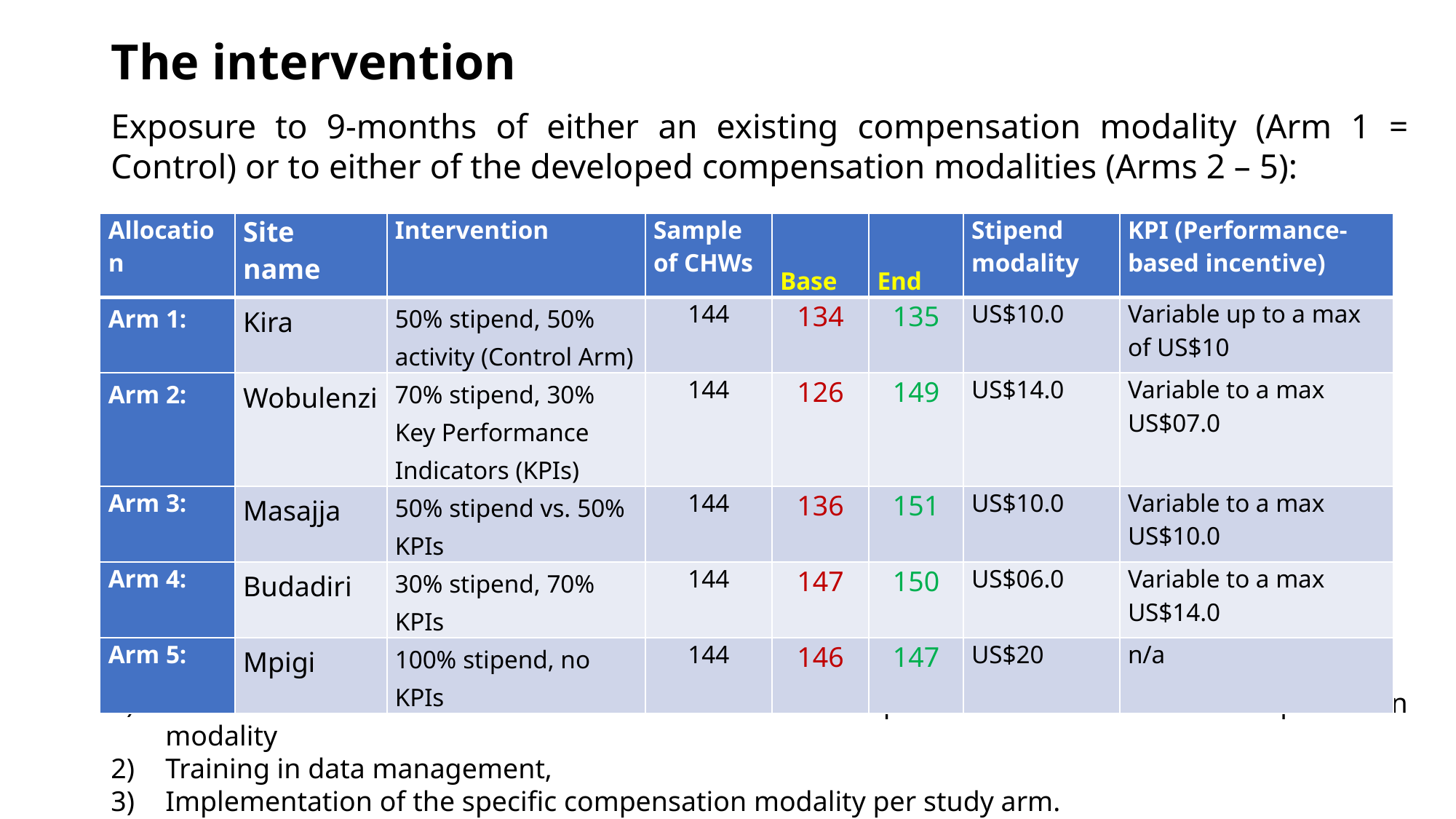

# The intervention
Exposure to 9-months of either an existing compensation modality (Arm 1 = Control) or to either of the developed compensation modalities (Arms 2 – 5):
Setting up the intervention comprised
One-month tailored orientation of the sites’ leadership and CHWs to the new compensation modality
Training in data management,
Implementation of the specific compensation modality per study arm.
| Allocation | Site name | Intervention | Sample of CHWs | Base | End | Stipend modality | KPI (Performance-based incentive) |
| --- | --- | --- | --- | --- | --- | --- | --- |
| Arm 1: | Kira | 50% stipend, 50% activity (Control Arm) | 144 | 134 | 135 | US$10.0 | Variable up to a max of US$10 |
| Arm 2: | Wobulenzi | 70% stipend, 30% Key Performance Indicators (KPIs) | 144 | 126 | 149 | US$14.0 | Variable to a max US$07.0 |
| Arm 3: | Masajja | 50% stipend vs. 50% KPIs | 144 | 136 | 151 | US$10.0 | Variable to a max US$10.0 |
| Arm 4: | Budadiri | 30% stipend, 70% KPIs | 144 | 147 | 150 | US$06.0 | Variable to a max US$14.0 |
| Arm 5: | Mpigi | 100% stipend, no KPIs | 144 | 146 | 147 | US$20 | n/a |

## Slide 11
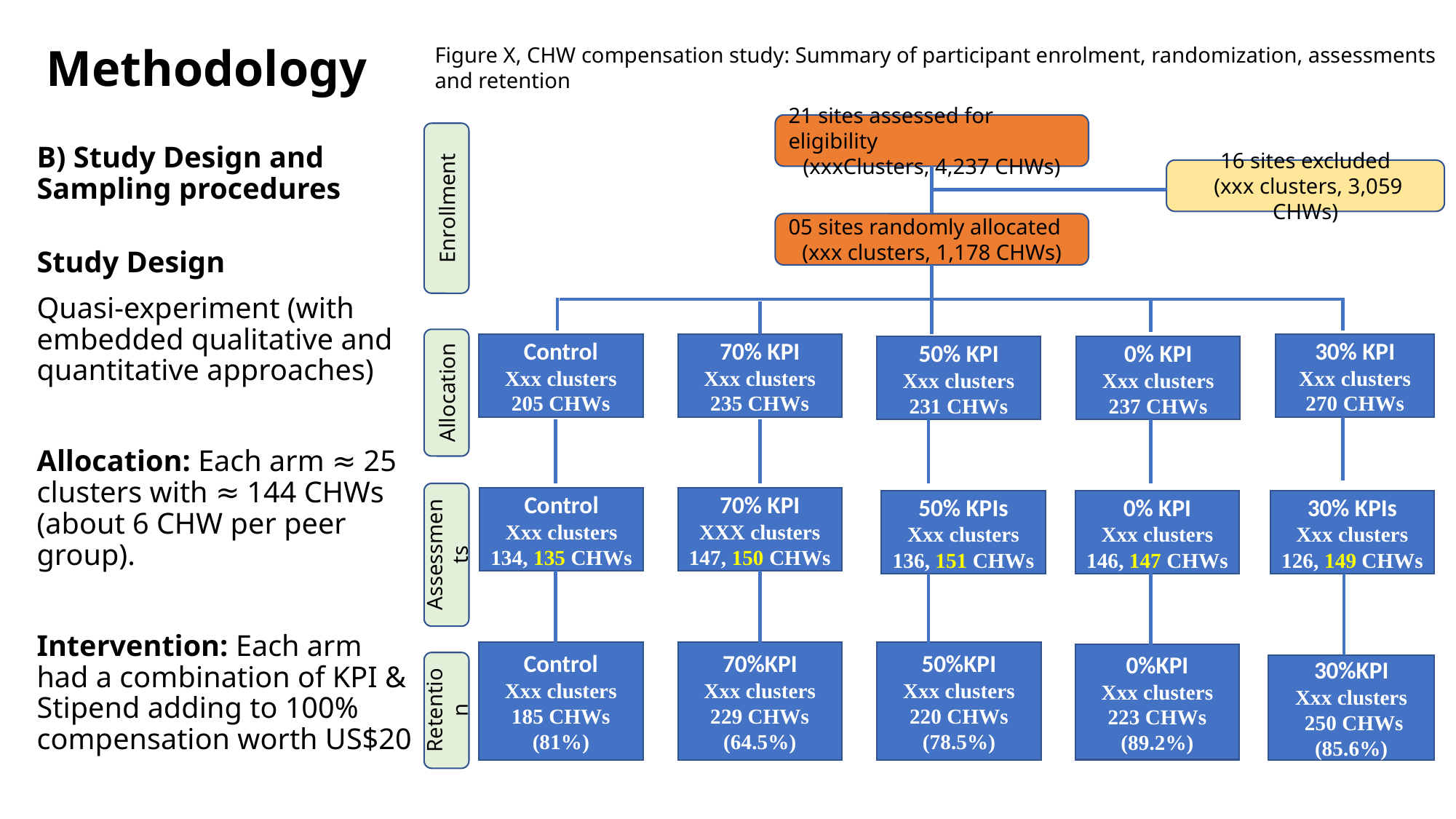

# Methodology
Figure X, CHW compensation study: Summary of participant enrolment, randomization, assessments and retention
Control
Xxx clusters
134, 135 CHWs
21 sites assessed for eligibility
(xxxClusters, 4,237 CHWs)
16 sites excluded
 (xxx clusters, 3,059 CHWs)
Enrollment
05 sites randomly allocated
(xxx clusters, 1,178 CHWs)
Control
Xxx clusters
205 CHWs
70% KPI
Xxx clusters
235 CHWs
30% KPI
Xxx clusters
270 CHWs
50% KPI
Xxx clusters
231 CHWs
0% KPI
Xxx clusters
237 CHWs
Allocation
70% KPI
XXX clusters
147, 150 CHWs
50% KPIs
Xxx clusters
136, 151 CHWs
0% KPI
Xxx clusters
146, 147 CHWs
30% KPIs
Xxx clusters
126, 149 CHWs
Assessments
Retention
70%KPI
Xxx clusters
229 CHWs
(64.5%)
Control
Xxx clusters
185 CHWs (81%)
50%KPI
Xxx clusters
220 CHWs
(78.5%)
0%KPI
Xxx clusters
223 CHWs
(89.2%)
30%KPI
Xxx clusters
 250 CHWs
(85.6%)
B) Study Design and Sampling procedures
Study Design
Quasi-experiment (with embedded qualitative and quantitative approaches)
Allocation: Each arm ≈ 25 clusters with ≈ 144 CHWs (about 6 CHW per peer group).
Intervention: Each arm had a combination of KPI & Stipend adding to 100% compensation worth US$20

## Slide 12
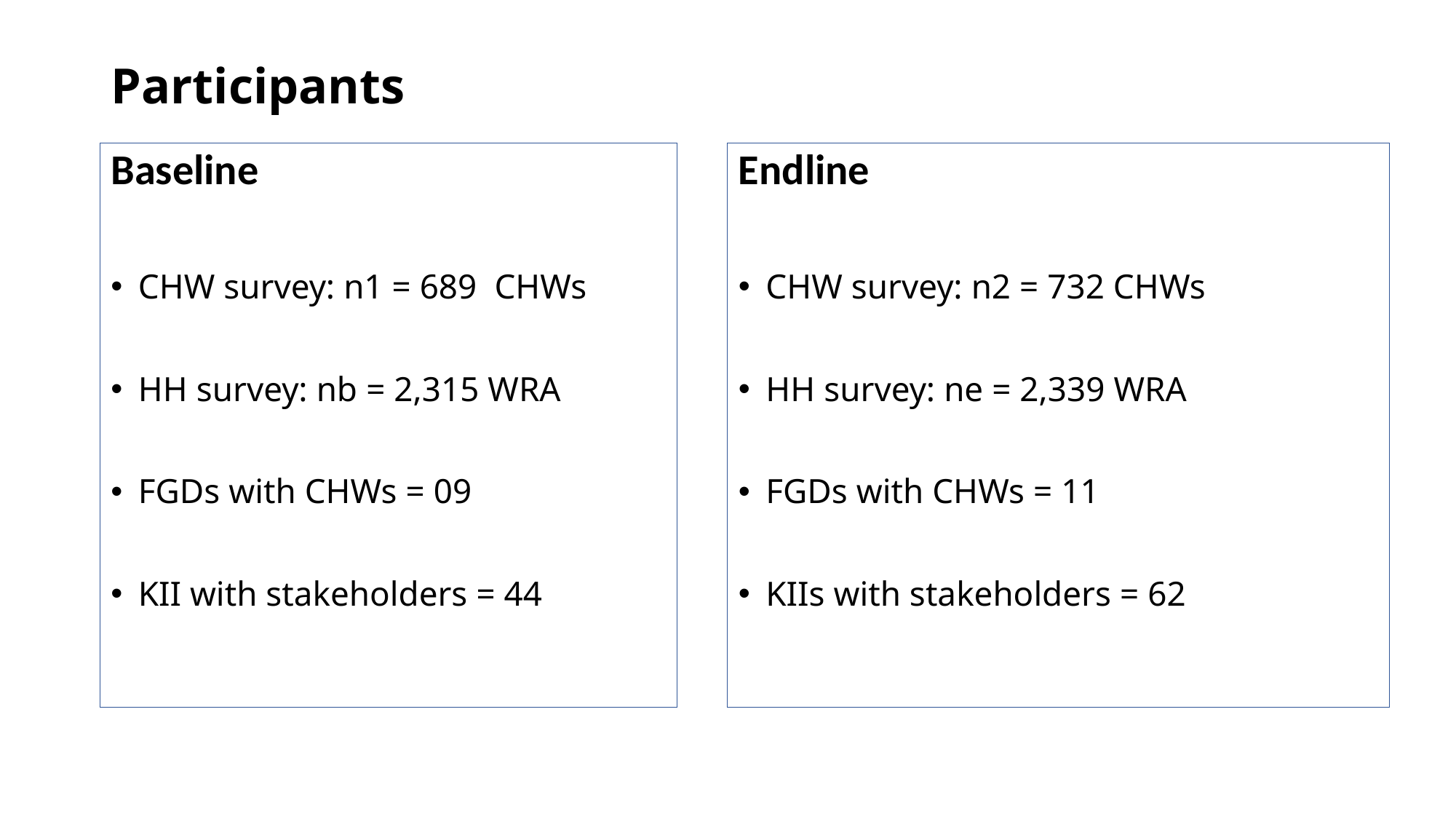

# Participants
Baseline
CHW survey: n1 = 689 CHWs
HH survey: nb = 2,315 WRA
FGDs with CHWs = 09
KII with stakeholders = 44
Endline
CHW survey: n2 = 732 CHWs
HH survey: ne = 2,339 WRA
FGDs with CHWs = 11
KIIs with stakeholders = 62

## Slide 13
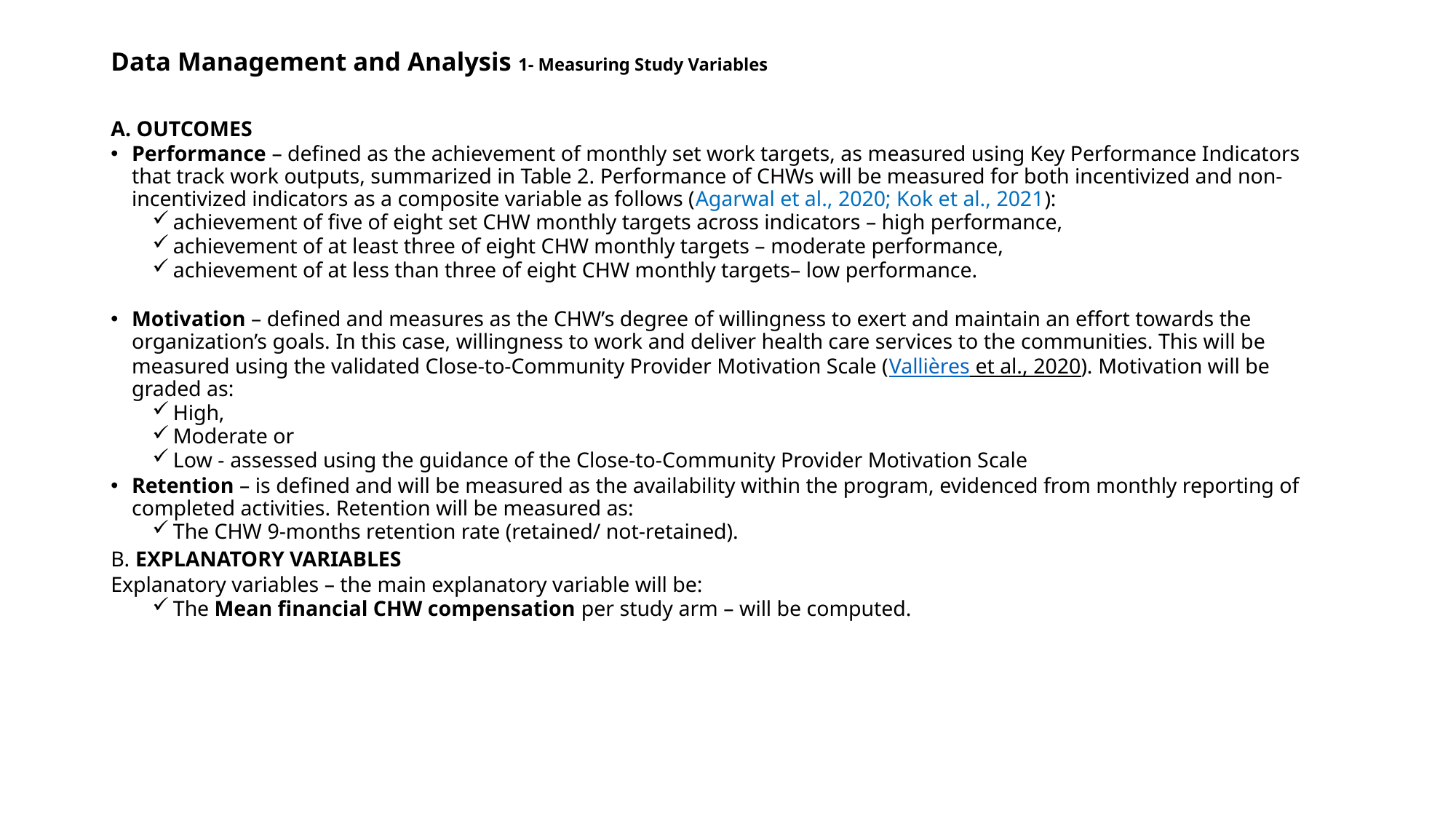

# Data Management and Analysis 1- Measuring Study Variables
A. OUTCOMES
Performance – defined as the achievement of monthly set work targets, as measured using Key Performance Indicators that track work outputs, summarized in Table 2. Performance of CHWs will be measured for both incentivized and non-incentivized indicators as a composite variable as follows (Agarwal et al., 2020; Kok et al., 2021):
achievement of five of eight set CHW monthly targets across indicators – high performance,
achievement of at least three of eight CHW monthly targets – moderate performance,
achievement of at less than three of eight CHW monthly targets– low performance.
Motivation – defined and measures as the CHW’s degree of willingness to exert and maintain an effort towards the organization’s goals. In this case, willingness to work and deliver health care services to the communities. This will be measured using the validated Close-to-Community Provider Motivation Scale (Vallières et al., 2020). Motivation will be graded as:
High,
Moderate or
Low - assessed using the guidance of the Close-to-Community Provider Motivation Scale
Retention – is defined and will be measured as the availability within the program, evidenced from monthly reporting of completed activities. Retention will be measured as:
The CHW 9-months retention rate (retained/ not-retained).
B. EXPLANATORY VARIABLES
Explanatory variables – the main explanatory variable will be:
The Mean financial CHW compensation per study arm – will be computed.

## Slide 14
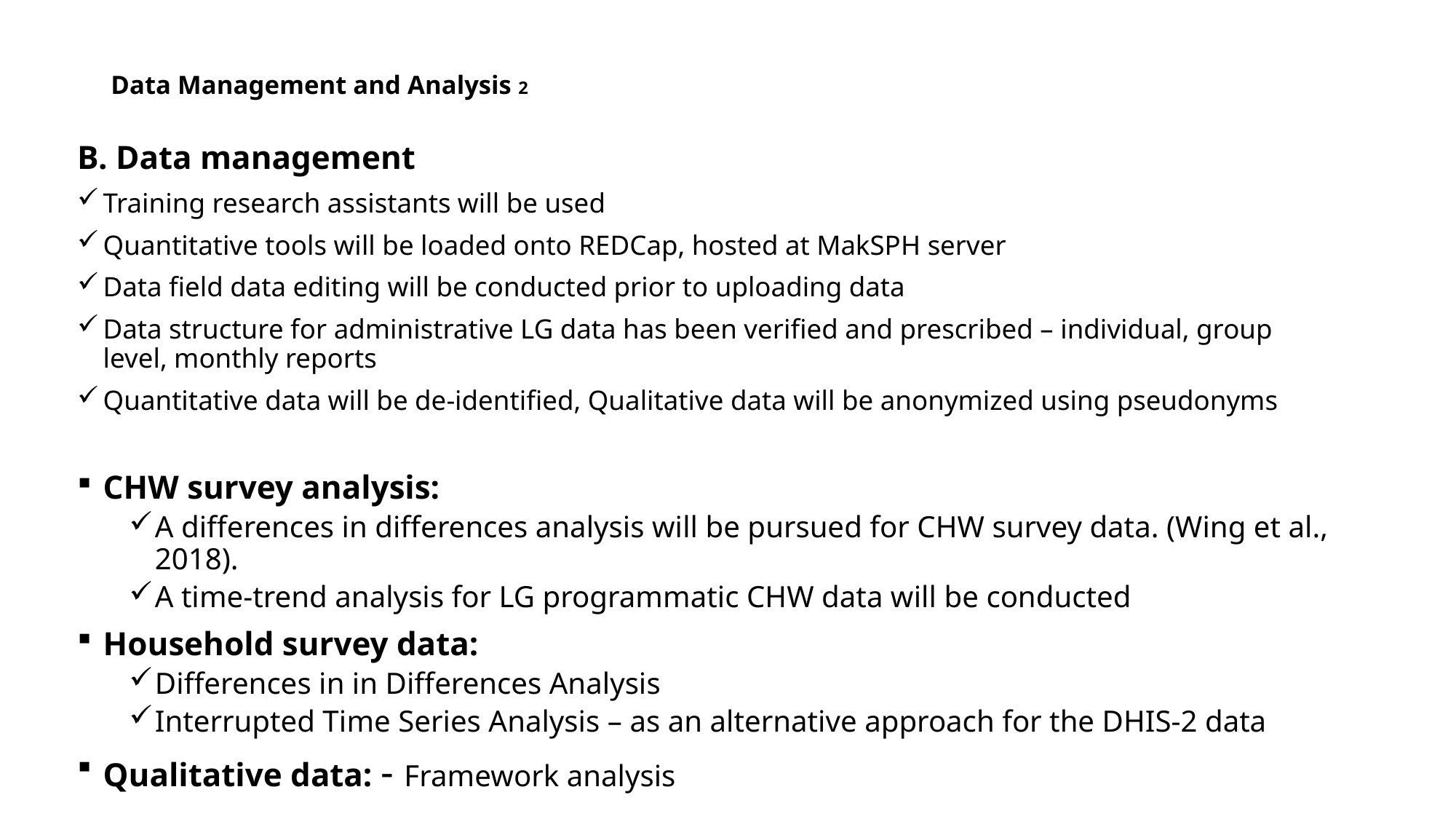

# Data Management and Analysis 2
B. Data management
Training research assistants will be used
Quantitative tools will be loaded onto REDCap, hosted at MakSPH server
Data field data editing will be conducted prior to uploading data
Data structure for administrative LG data has been verified and prescribed – individual, group level, monthly reports
Quantitative data will be de-identified, Qualitative data will be anonymized using pseudonyms
CHW survey analysis:
A differences in differences analysis will be pursued for CHW survey data. (Wing et al., 2018).
A time-trend analysis for LG programmatic CHW data will be conducted
Household survey data:
Differences in in Differences Analysis
Interrupted Time Series Analysis – as an alternative approach for the DHIS-2 data
Qualitative data: - Framework analysis

## Slide 15
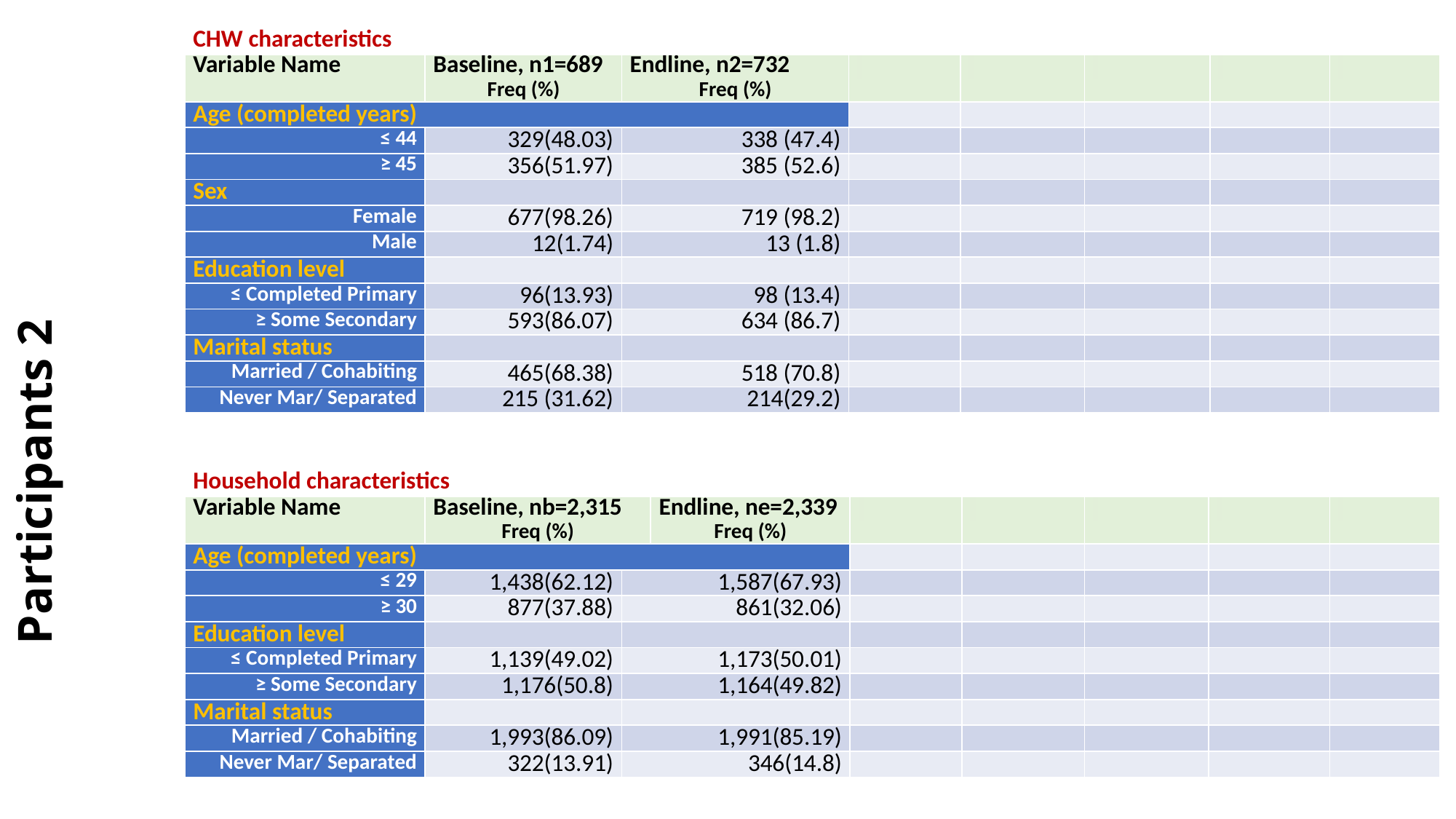

| CHW characteristics | | | | | | | |
| --- | --- | --- | --- | --- | --- | --- | --- |
| Variable Name | Baseline, n1=689 Freq (%) | Endline, n2=732 Freq (%) | | | | | |
| Age (completed years) | | | | | | | |
| ≤ 44 | 329(48.03) | 338 (47.4) | | | | | |
| ≥ 45 | 356(51.97) | 385 (52.6) | | | | | |
| Sex | | | | | | | |
| Female | 677(98.26) | 719 (98.2) | | | | | |
| Male | 12(1.74) | 13 (1.8) | | | | | |
| Education level | | | | | | | |
| ≤ Completed Primary | 96(13.93) | 98 (13.4) | | | | | |
| ≥ Some Secondary | 593(86.07) | 634 (86.7) | | | | | |
| Marital status | | | | | | | |
| Married / Cohabiting | 465(68.38) | 518 (70.8) | | | | | |
| Never Mar/ Separated | 215 (31.62) | 214(29.2) | | | | | |
# Participants 2
| Household characteristics | | | | | | | | |
| --- | --- | --- | --- | --- | --- | --- | --- | --- |
| Variable Name | Baseline, nb=2,315 Freq (%) | Endline, ne=2,339 Freq (%) | Endline, ne=2,339 Freq (%) | | | | | |
| Age (completed years) | | | | | | | | |
| ≤ 29 | 1,438(62.12) | 1,587(67.93) | | | | | | |
| ≥ 30 | 877(37.88) | 861(32.06) | | | | | | |
| Education level | | | | | | | | |
| ≤ Completed Primary | 1,139(49.02) | 1,173(50.01) | | | | | | |
| ≥ Some Secondary | 1,176(50.8) | 1,164(49.82) | | | | | | |
| Marital status | | | | | | | | |
| Married / Cohabiting | 1,993(86.09) | 1,991(85.19) | | | | | | |
| Never Mar/ Separated | 322(13.91) | 346(14.8) | | | | | | |

## Slide 16
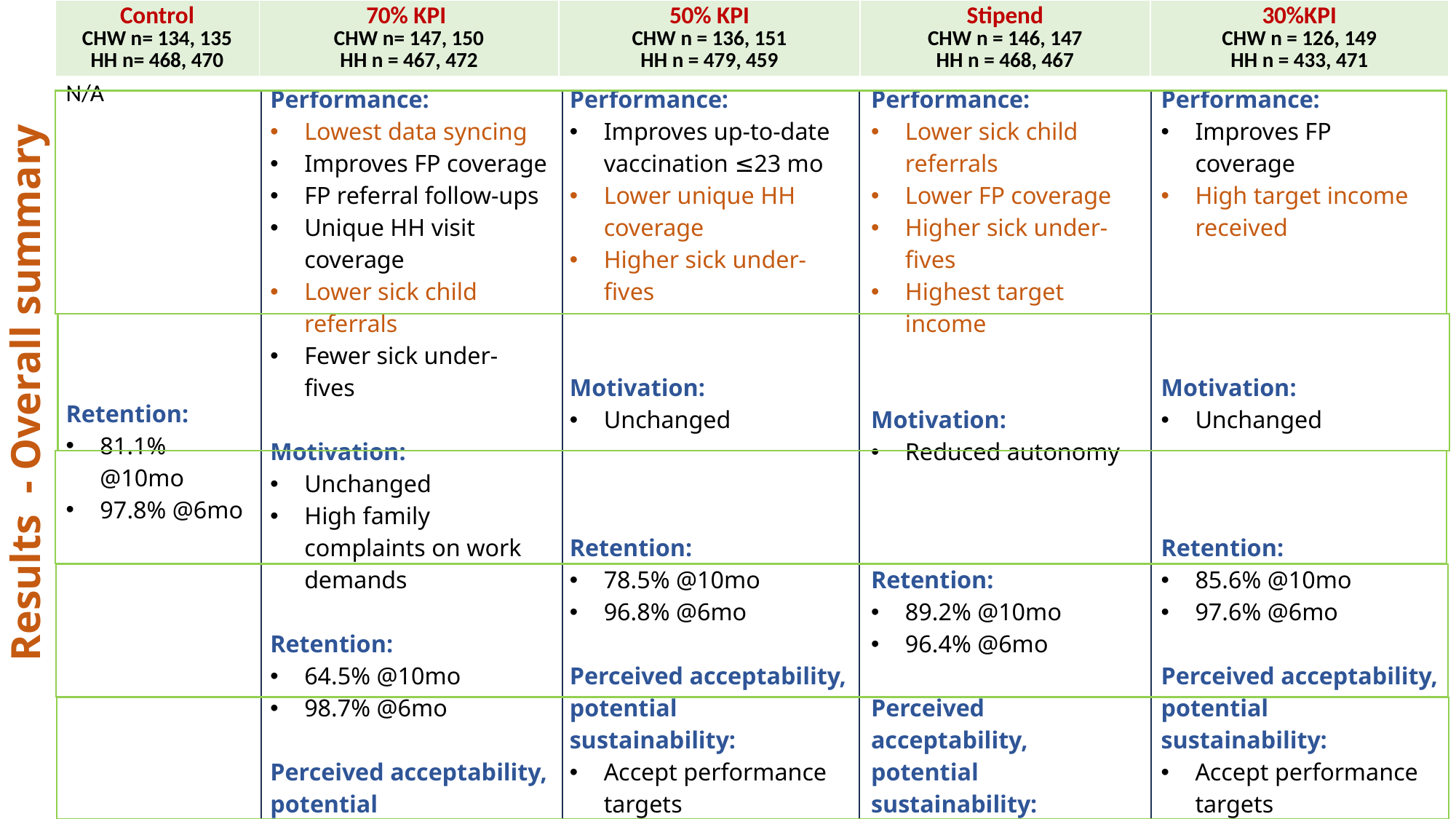

| Control CHW n= 134, 135 HH n= 468, 470 | 70% KPI CHW n= 147, 150 HH n = 467, 472 | 50% KPI CHW n = 136, 151 HH n = 479, 459 | Stipend CHW n = 146, 147 HH n = 468, 467 | 30%KPI CHW n = 126, 149 HH n = 433, 471 |
| --- | --- | --- | --- | --- |
| N/A Retention: 81.1% @10mo 97.8% @6mo | Performance: Lowest data syncing Improves FP coverage FP referral follow-ups Unique HH visit coverage Lower sick child referrals Fewer sick under-fives Motivation: Unchanged High family complaints on work demands Retention: 64.5% @10mo 98.7% @6mo Perceived acceptability, potential sustainability: Unfair performance targets Impact to personal income and livelihood: High | Performance: Improves up-to-date vaccination ≤23 mo Lower unique HH coverage Higher sick under-fives Motivation: Unchanged Retention: 78.5% @10mo 96.8% @6mo Perceived acceptability, potential sustainability: Accept performance targets Impact to personal income and livelihood: High | Performance: Lower sick child referrals Lower FP coverage Higher sick under-fives Highest target income Motivation: Reduced autonomy Retention: 89.2% @10mo 96.4% @6mo Perceived acceptability, potential sustainability: Accept performance targets Impact to personal income and livelihood: High | Performance: Improves FP coverage High target income received Motivation: Unchanged Retention: 85.6% @10mo 97.6% @6mo Perceived acceptability, potential sustainability: Accept performance targets Impact to personal income and livelihood: High |
# Results - Overall summary

## Slide 17
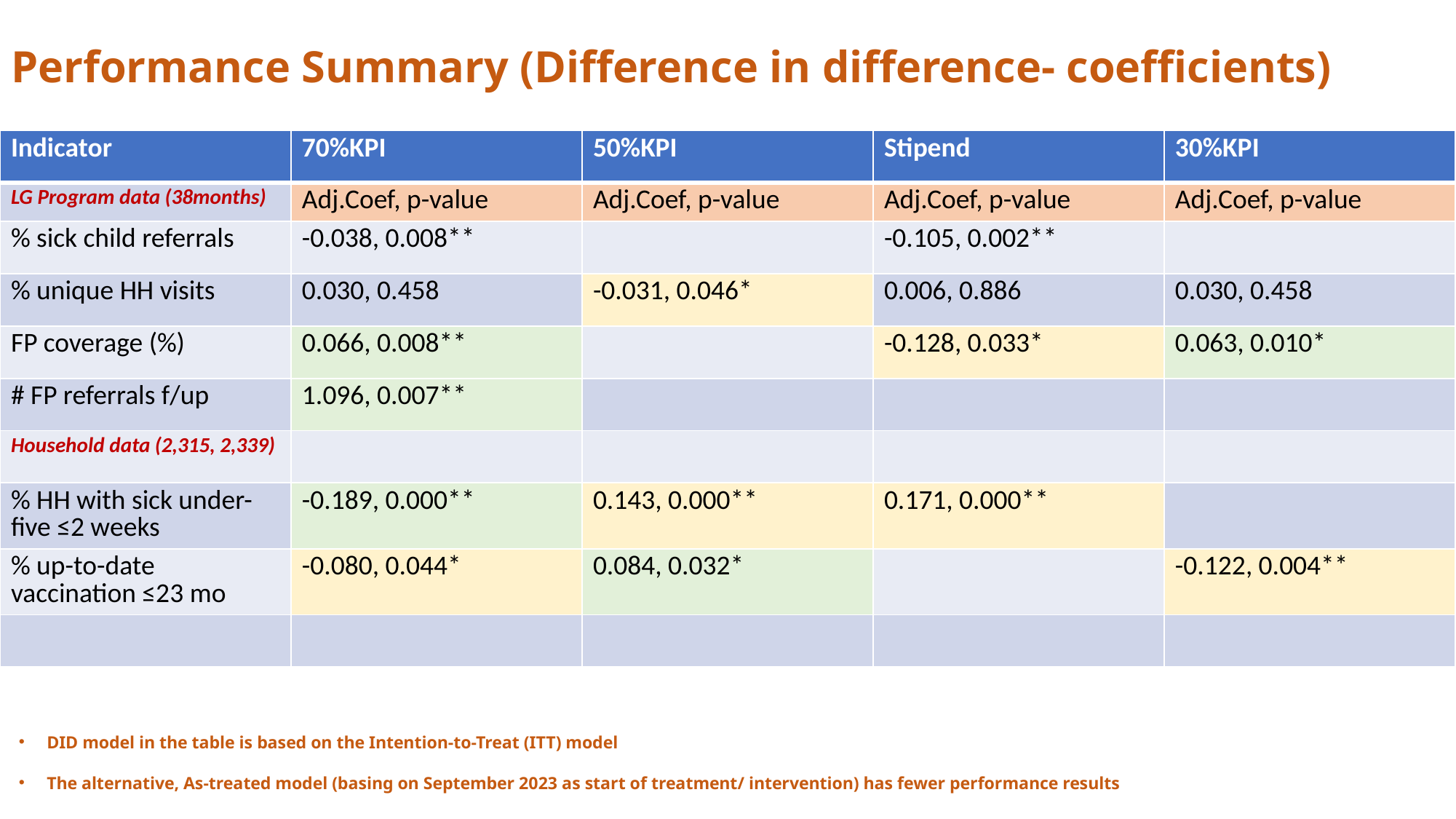

# Performance Summary (Difference in difference- coefficients)
| Indicator | 70%KPI | 50%KPI | Stipend | 30%KPI |
| --- | --- | --- | --- | --- |
| LG Program data (38months) | Adj.Coef, p-value | Adj.Coef, p-value | Adj.Coef, p-value | Adj.Coef, p-value |
| % sick child referrals | -0.038, 0.008\*\* | | -0.105, 0.002\*\* | |
| % unique HH visits | 0.030, 0.458 | -0.031, 0.046\* | 0.006, 0.886 | 0.030, 0.458 |
| FP coverage (%) | 0.066, 0.008\*\* | | -0.128, 0.033\* | 0.063, 0.010\* |
| # FP referrals f/up | 1.096, 0.007\*\* | | | |
| Household data (2,315, 2,339) | | | | |
| % HH with sick under-five ≤2 weeks | -0.189, 0.000\*\* | 0.143, 0.000\*\* | 0.171, 0.000\*\* | |
| % up-to-date vaccination ≤23 mo | -0.080, 0.044\* | 0.084, 0.032\* | | -0.122, 0.004\*\* |
| | | | | |
DID model in the table is based on the Intention-to-Treat (ITT) model
The alternative, As-treated model (basing on September 2023 as start of treatment/ intervention) has fewer performance results

## Slide 18
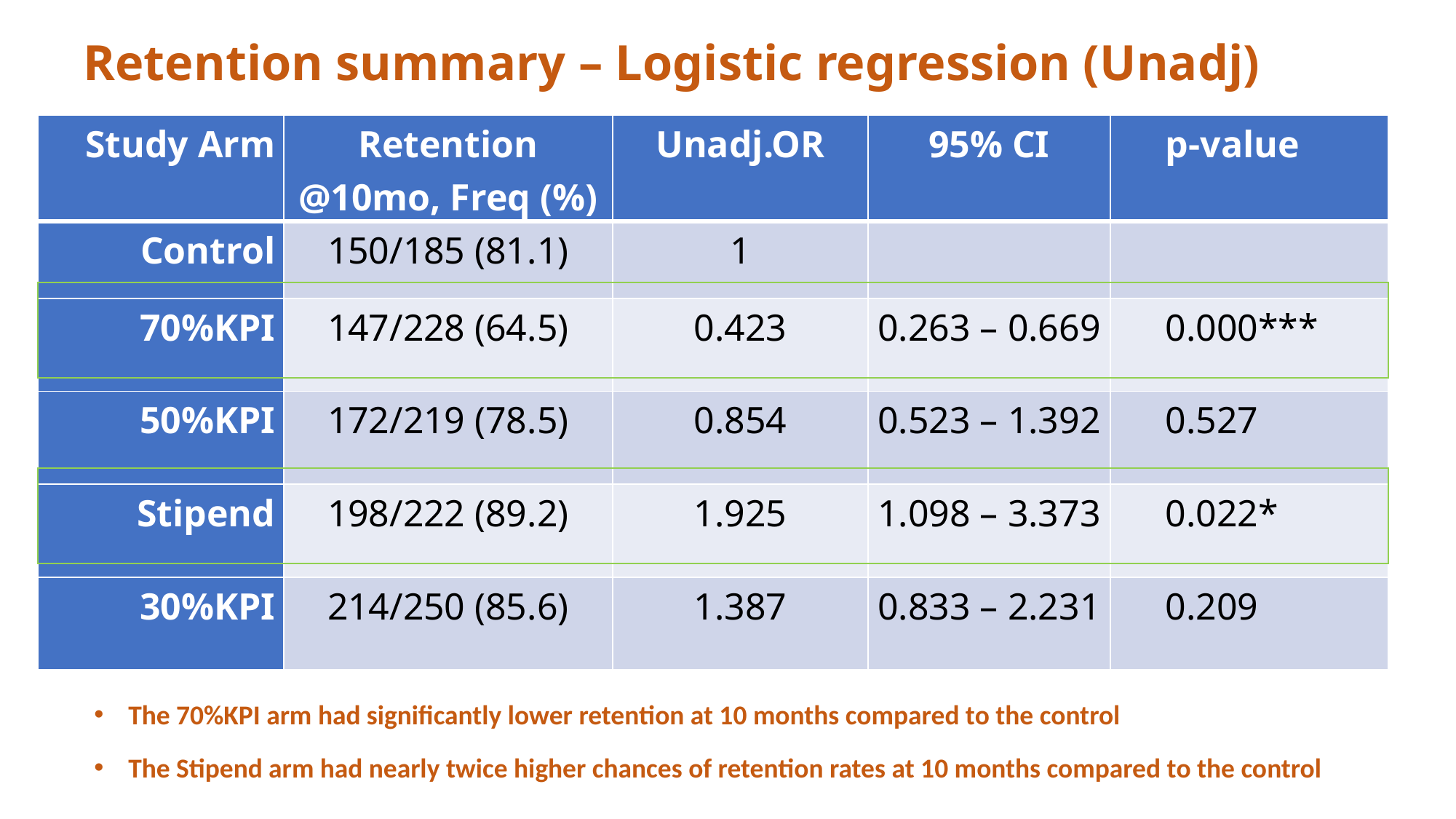

# Retention summary – Logistic regression (Unadj)
| Study Arm | Retention @10mo, Freq (%) | Unadj.OR | 95% CI | p-value |
| --- | --- | --- | --- | --- |
| Control | 150/185 (81.1) | 1 | | |
| 70%KPI | 147/228 (64.5) | 0.423 | 0.263 – 0.669 | 0.000\*\*\* |
| 50%KPI | 172/219 (78.5) | 0.854 | 0.523 – 1.392 | 0.527 |
| Stipend | 198/222 (89.2) | 1.925 | 1.098 – 3.373 | 0.022\* |
| 30%KPI | 214/250 (85.6) | 1.387 | 0.833 – 2.231 | 0.209 |
The 70%KPI arm had significantly lower retention at 10 months compared to the control
The Stipend arm had nearly twice higher chances of retention rates at 10 months compared to the control

## Slide 19
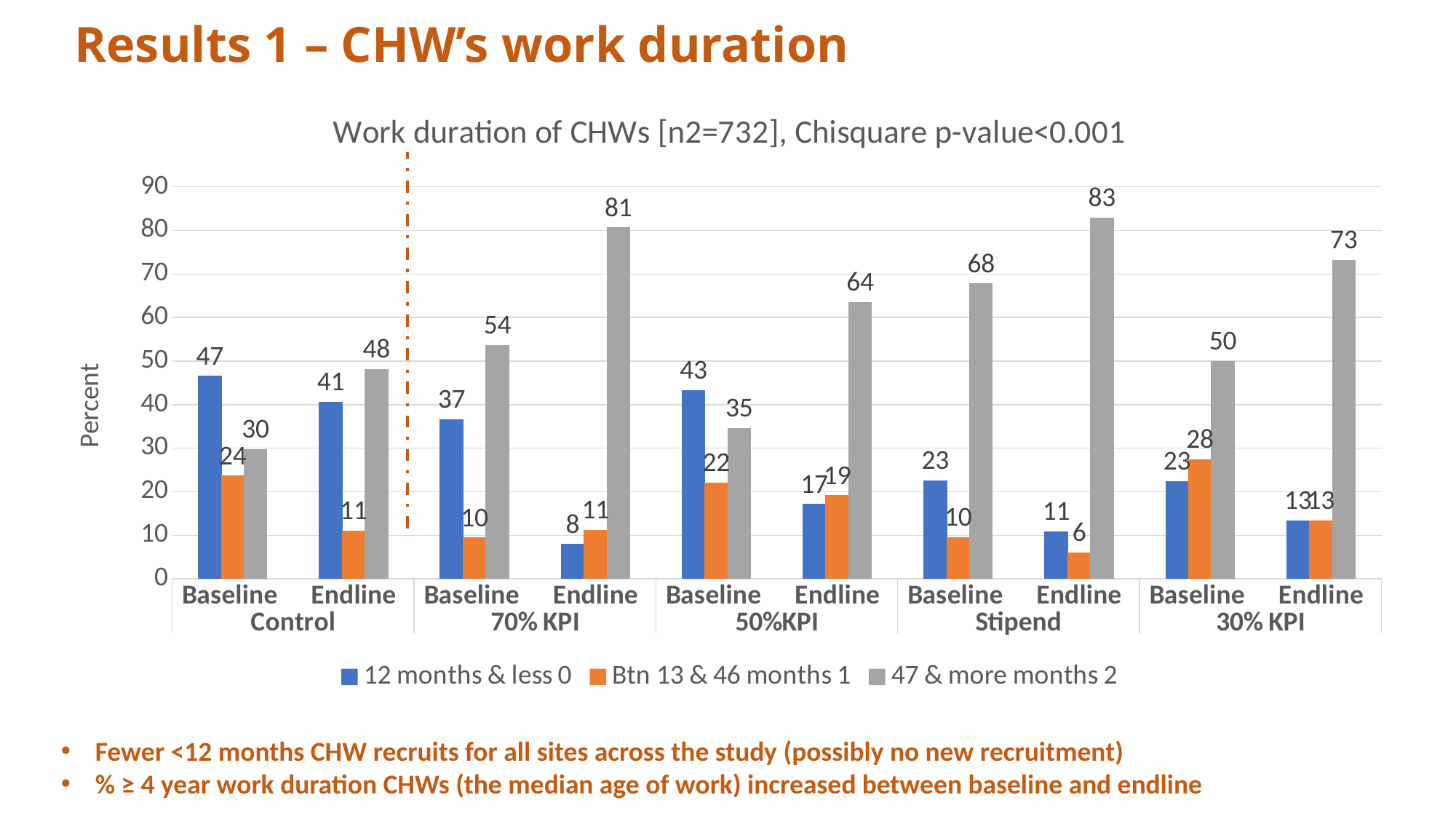

# Results 1 – CHW’s work duration
### Chart: Work duration of CHWs [n2=732], Chisquare p-value<0.001
| Category | 12 months & less 0 | Btn 13 & 46 months 1 | 47 & more months 2 |
|---|---|---|---|
| Baseline | 46.6 | 23.7 | 29.8 |
| Endline | 40.7 | 11.1 | 48.2 |
| Baseline | 36.7 | 9.5 | 53.7 |
| Endline | 8.0 | 11.3 | 80.7 |
| Baseline | 43.4 | 22.1 | 34.6 |
| Endline | 17.2 | 19.2 | 63.6 |
| Baseline | 22.6 | 9.6 | 67.8 |
| Endline | 10.9 | 6.1 | 83.0 |
| Baseline | 22.5 | 27.5 | 50.0 |
| Endline | 13.4 | 13.4 | 73.2 |Fewer <12 months CHW recruits for all sites across the study (possibly no new recruitment)
% ≥ 4 year work duration CHWs (the median age of work) increased between baseline and endline

## Slide 20
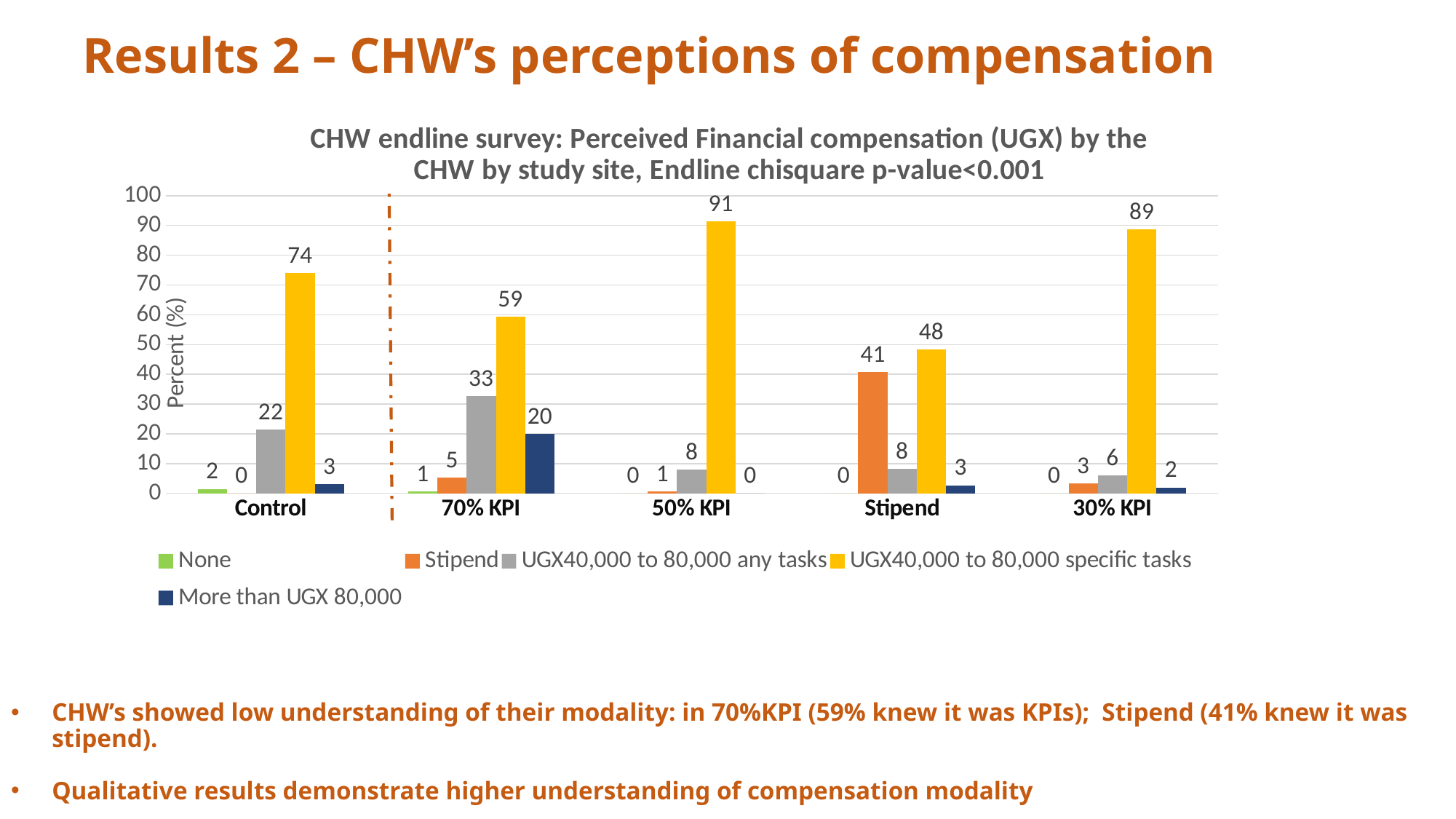

# Results 2 – CHW’s perceptions of compensation
### Chart: CHW endline survey: Perceived Financial compensation (UGX) by the CHW by study site, Endline chisquare p-value<0.001
| Category | None | Stipend | UGX40,000 to 80,000 any tasks | UGX40,000 to 80,000 specific tasks | More than UGX 80,000 |
|---|---|---|---|---|---|
| Control | 1.5 | 0.0 | 21.5 | 74.10000000000001 | 3.0 |
| 70% KPI | 0.7 | 5.3 | 32.7 | 59.3 | 20.0 |
| 50% KPI | 0.0 | 0.7 | 8.0 | 91.4 | 0.0 |
| Stipend | 0.0 | 40.8 | 8.2 | 48.3 | 2.7 |
| 30% KPI | 0.0 | 3.4 | 6.0 | 88.6 | 2.0 |CHW’s showed low understanding of their modality: in 70%KPI (59% knew it was KPIs); Stipend (41% knew it was stipend).
Qualitative results demonstrate higher understanding of compensation modality

## Slide 21
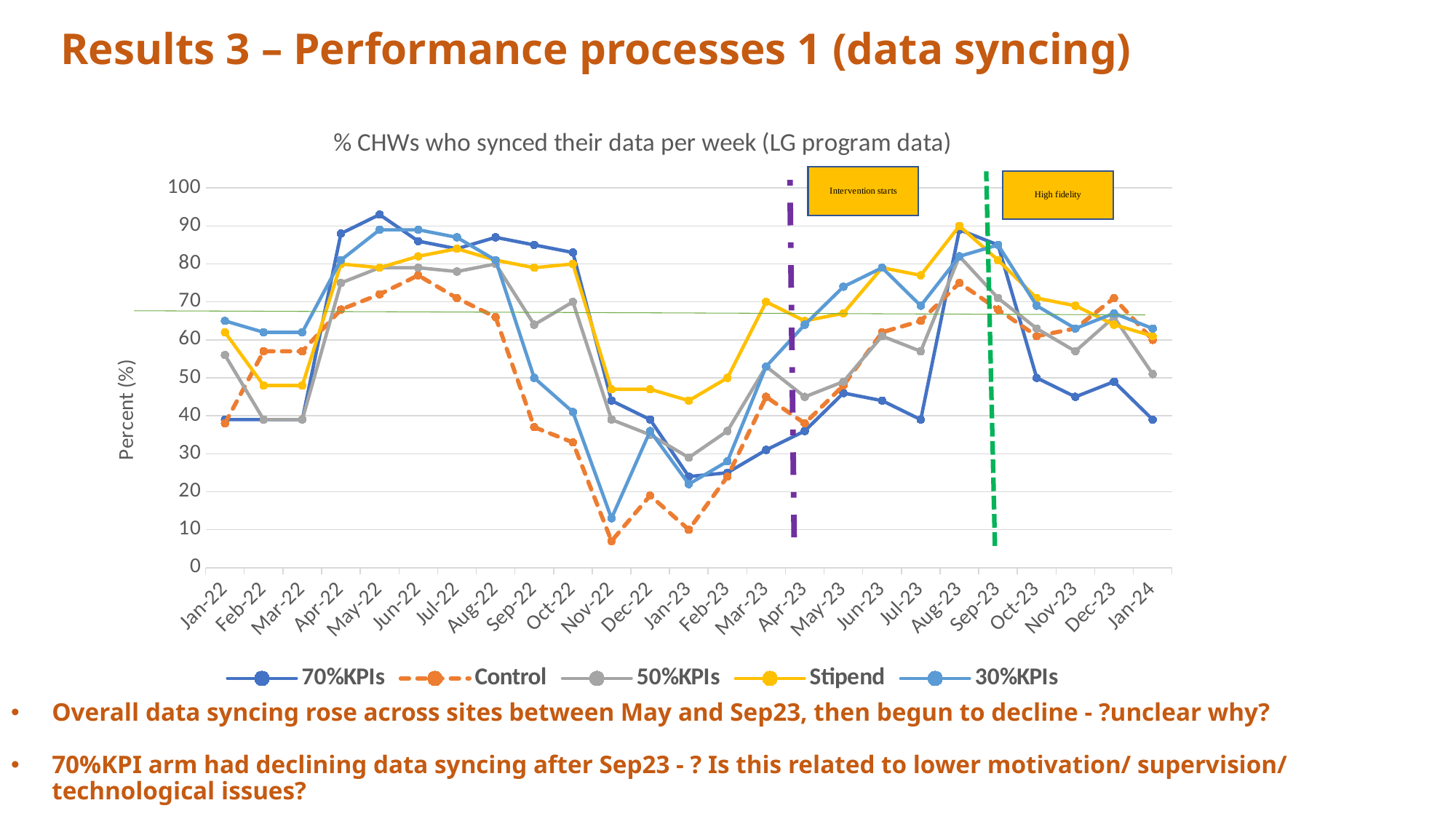

# Results 3 – Performance processes 1 (data syncing)
### Chart: % CHWs who synced their data per week (LG program data)
| Category | 70%KPIs | Control | 50%KPIs | Stipend | 30%KPIs |
|---|---|---|---|---|---|
| 44562 | 39.0 | 38.0 | 56.0 | 62.0 | 65.0 |
| 44593 | 39.0 | 57.0 | 39.0 | 48.0 | 62.0 |
| 44621 | 39.0 | 57.0 | 39.0 | 48.0 | 62.0 |
| 44652 | 88.0 | 68.0 | 75.0 | 80.0 | 81.0 |
| 44682 | 93.0 | 72.0 | 79.0 | 79.0 | 89.0 |
| 44713 | 86.0 | 77.0 | 79.0 | 82.0 | 89.0 |
| 44743 | 84.0 | 71.0 | 78.0 | 84.0 | 87.0 |
| 44774 | 87.0 | 66.0 | 80.0 | 81.0 | 81.0 |
| 44805 | 85.0 | 37.0 | 64.0 | 79.0 | 50.0 |
| 44835 | 83.0 | 33.0 | 70.0 | 80.0 | 41.0 |
| 44866 | 44.0 | 7.0 | 39.0 | 47.0 | 13.0 |
| 44896 | 39.0 | 19.0 | 35.0 | 47.0 | 36.0 |
| 44927 | 24.0 | 10.0 | 29.0 | 44.0 | 22.0 |
| 44958 | 25.0 | 24.0 | 36.0 | 50.0 | 28.0 |
| 44986 | 31.0 | 45.0 | 53.0 | 70.0 | 53.0 |
| 45017 | 36.0 | 38.0 | 45.0 | 65.0 | 64.0 |
| 45047 | 46.0 | 48.0 | 49.0 | 67.0 | 74.0 |
| 45078 | 44.0 | 62.0 | 61.0 | 79.0 | 79.0 |
| 45108 | 39.0 | 65.0 | 57.0 | 77.0 | 69.0 |
| 45139 | 89.0 | 75.0 | 82.0 | 90.0 | 82.0 |
| 45170 | 85.0 | 68.0 | 71.0 | 81.0 | 85.0 |
| 45200 | 50.0 | 61.0 | 63.0 | 71.0 | 69.0 |
| 45231 | 45.0 | 63.0 | 57.0 | 69.0 | 63.0 |
| 45261 | 49.0 | 71.0 | 66.0 | 64.0 | 67.0 |
| 45292 | 39.0 | 60.0 | 51.0 | 61.0 | 63.0 |
| | None | None | None | None | None |Overall data syncing rose across sites between May and Sep23, then begun to decline - ?unclear why?
70%KPI arm had declining data syncing after Sep23 - ? Is this related to lower motivation/ supervision/ technological issues?

## Slide 22
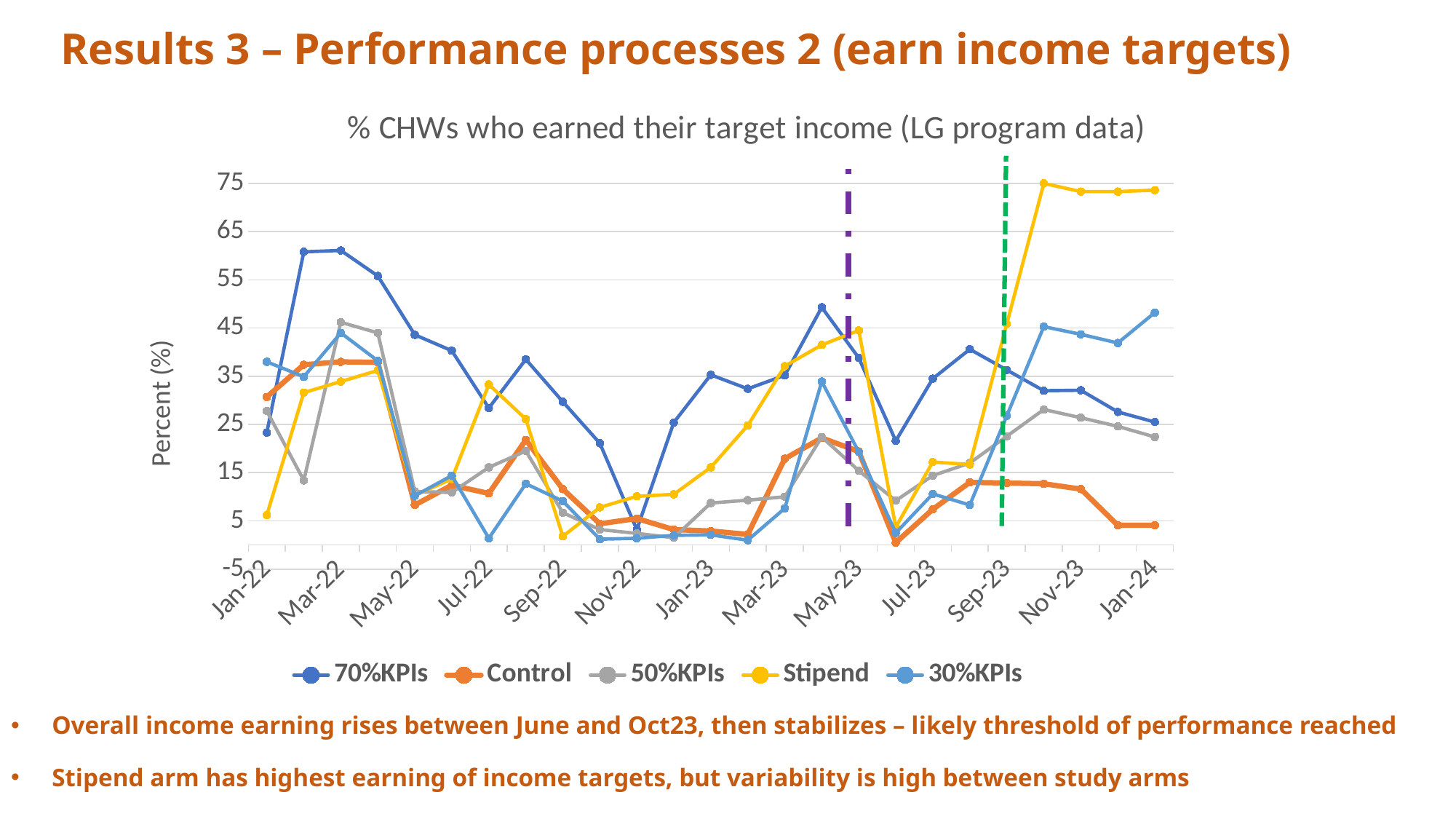

# Results 3 – Performance processes 2 (earn income targets)
### Chart: % CHWs who earned their target income (LG program data)
| Category | 70%KPIs | Control | 50%KPIs | Stipend | 30%KPIs |
|---|---|---|---|---|---|
| 44562 | 23.3 | 30.7 | 27.8 | 6.2 | 38.0 |
| 44593 | 60.8 | 37.4 | 13.4 | 31.6 | 34.9 |
| 44621 | 61.1 | 38.0 | 46.2 | 33.9 | 44.0 |
| 44652 | 55.8 | 37.9 | 44.0 | 36.2 | 38.2 |
| 44682 | 43.6 | 8.3 | 11.1 | 10.3 | 10.2 |
| 44713 | 40.3 | 12.4 | 10.9 | 13.7 | 14.4 |
| 44743 | 28.4 | 10.7 | 16.1 | 33.3 | 1.4 |
| 44774 | 38.5 | 21.8 | 19.5 | 26.1 | 12.7 |
| 44805 | 29.7 | 11.6 | 6.7 | 1.8 | 9.1 |
| 44835 | 21.1 | 4.4 | 3.2 | 7.8 | 1.2 |
| 44866 | 3.2 | 5.5 | 2.4 | 10.1 | 1.4 |
| 44896 | 25.4 | 3.2 | 1.5 | 10.5 | 2.0 |
| 44927 | 35.3 | 2.9 | 8.7 | 16.1 | 2.1 |
| 44958 | 32.4 | 2.2 | 9.3 | 24.8 | 1.0 |
| 44986 | 35.2 | 17.9 | 10.0 | 37.1 | 7.6 |
| 45017 | 49.3 | 22.2 | 22.4 | 41.5 | 33.9 |
| 45047 | 38.8 | 19.4 | 15.4 | 44.5 | 19.3 |
| 45078 | 21.6 | 0.5 | 9.2 | 3.7 | 2.5 |
| 45108 | 34.5 | 7.4 | 14.4 | 17.2 | 10.6 |
| 45139 | 40.6 | 13.0 | 17.0 | 16.7 | 8.3 |
| 45170 | 36.3 | 12.85 | 22.55 | 45.85 | 26.799999999999997 |
| 45200 | 32.0 | 12.7 | 28.1 | 75.0 | 45.3 |
| 45231 | 32.1 | 11.6 | 26.4 | 73.3 | 43.7 |
| 45261 | 27.6 | 4.1 | 24.6 | 73.3 | 41.9 |
| 45292 | 25.5 | 4.1 | 22.4 | 73.6 | 48.2 |
| | None | None | None | None | None |Overall income earning rises between June and Oct23, then stabilizes – likely threshold of performance reached
Stipend arm has highest earning of income targets, but variability is high between study arms

## Slide 23
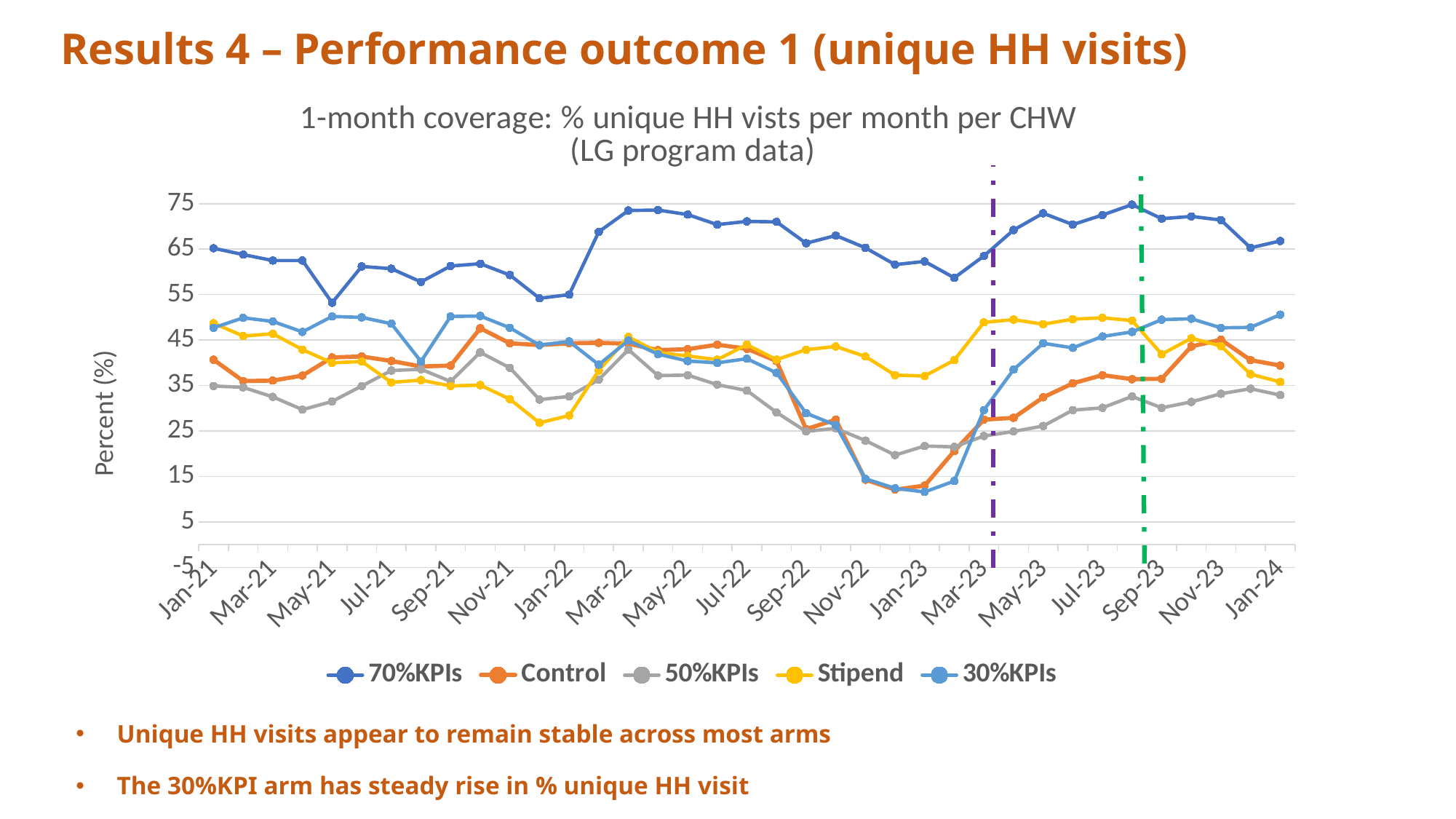

# Results 4 – Performance outcome 1 (unique HH visits)
### Chart: 1-month coverage: % unique HH vists per month per CHW
(LG program data)
| Category | 70%KPIs | Control | 50%KPIs | Stipend | 30%KPIs |
|---|---|---|---|---|---|
| 44197 | 65.2 | 40.7 | 34.9 | 48.7 | 47.7 |
| 44228 | 63.8 | 36.0 | 34.6 | 45.9 | 49.9 |
| 44256 | 62.5 | 36.1 | 32.5 | 46.4 | 49.1 |
| 44287 | 62.5 | 37.2 | 29.7 | 42.9 | 46.8 |
| 44317 | 53.2 | 41.2 | 31.5 | 40.0 | 50.2 |
| 44348 | 61.2 | 41.4 | 34.9 | 40.3 | 50.0 |
| 44378 | 60.7 | 40.4 | 38.3 | 35.7 | 48.6 |
| 44409 | 57.8 | 39.2 | 38.6 | 36.2 | 40.3 |
| 44440 | 61.3 | 39.4 | 35.9 | 34.9 | 50.2 |
| 44470 | 61.8 | 47.6 | 42.3 | 35.1 | 50.3 |
| 44501 | 59.3 | 44.3 | 38.9 | 32.0 | 47.7 |
| 44531 | 54.2 | 43.9 | 31.9 | 26.8 | 43.9 |
| 44562 | 55.0 | 44.3 | 32.6 | 28.4 | 44.7 |
| 44593 | 68.8 | 44.4 | 36.3 | 38.3 | 39.6 |
| 44621 | 73.5 | 44.2 | 42.9 | 45.7 | 44.9 |
| 44652 | 73.6 | 42.8 | 37.2 | 42.3 | 41.9 |
| 44682 | 72.6 | 43.0 | 37.3 | 41.5 | 40.4 |
| 44713 | 70.4 | 44.0 | 35.2 | 40.7 | 40.0 |
| 44743 | 71.1 | 43.1 | 33.9 | 44.0 | 40.9 |
| 44774 | 71.0 | 40.4 | 29.1 | 40.7 | 37.8 |
| 44805 | 66.3 | 25.4 | 24.9 | 42.9 | 28.9 |
| 44835 | 68.0 | 27.5 | 25.6 | 43.6 | 26.3 |
| 44866 | 65.3 | 14.3 | 22.9 | 41.4 | 14.5 |
| 44896 | 61.6 | 12.1 | 19.7 | 37.3 | 12.4 |
| 44927 | 62.3 | 13.0 | 21.7 | 37.1 | 11.6 |
| 44958 | 58.7 | 20.6 | 21.5 | 40.6 | 14.0 |
| 44986 | 63.5 | 27.5 | 23.9 | 48.9 | 29.6 |
| 45017 | 69.2 | 27.9 | 24.9 | 49.5 | 38.5 |
| 45047 | 72.9 | 32.4 | 26.1 | 48.5 | 44.3 |
| 45078 | 70.4 | 35.5 | 29.6 | 49.6 | 43.3 |
| 45108 | 72.5 | 37.3 | 30.1 | 49.9 | 45.8 |
| 45139 | 74.8 | 36.4 | 32.6 | 49.3 | 46.8 |
| 45170 | 71.7 | 36.5 | 30.1 | 41.9 | 49.5 |
| 45200 | 72.2 | 43.6 | 31.4 | 45.4 | 49.7 |
| 45231 | 71.4 | 45.1 | 33.2 | 43.7 | 47.7 |
| 45261 | 65.3 | 40.6 | 34.3 | 37.5 | 47.8 |
| 45292 | 66.8 | 39.4 | 32.9 | 35.8 | 50.6 |
| | None | None | None | None | None |Unique HH visits appear to remain stable across most arms
The 30%KPI arm has steady rise in % unique HH visit

## Slide 24
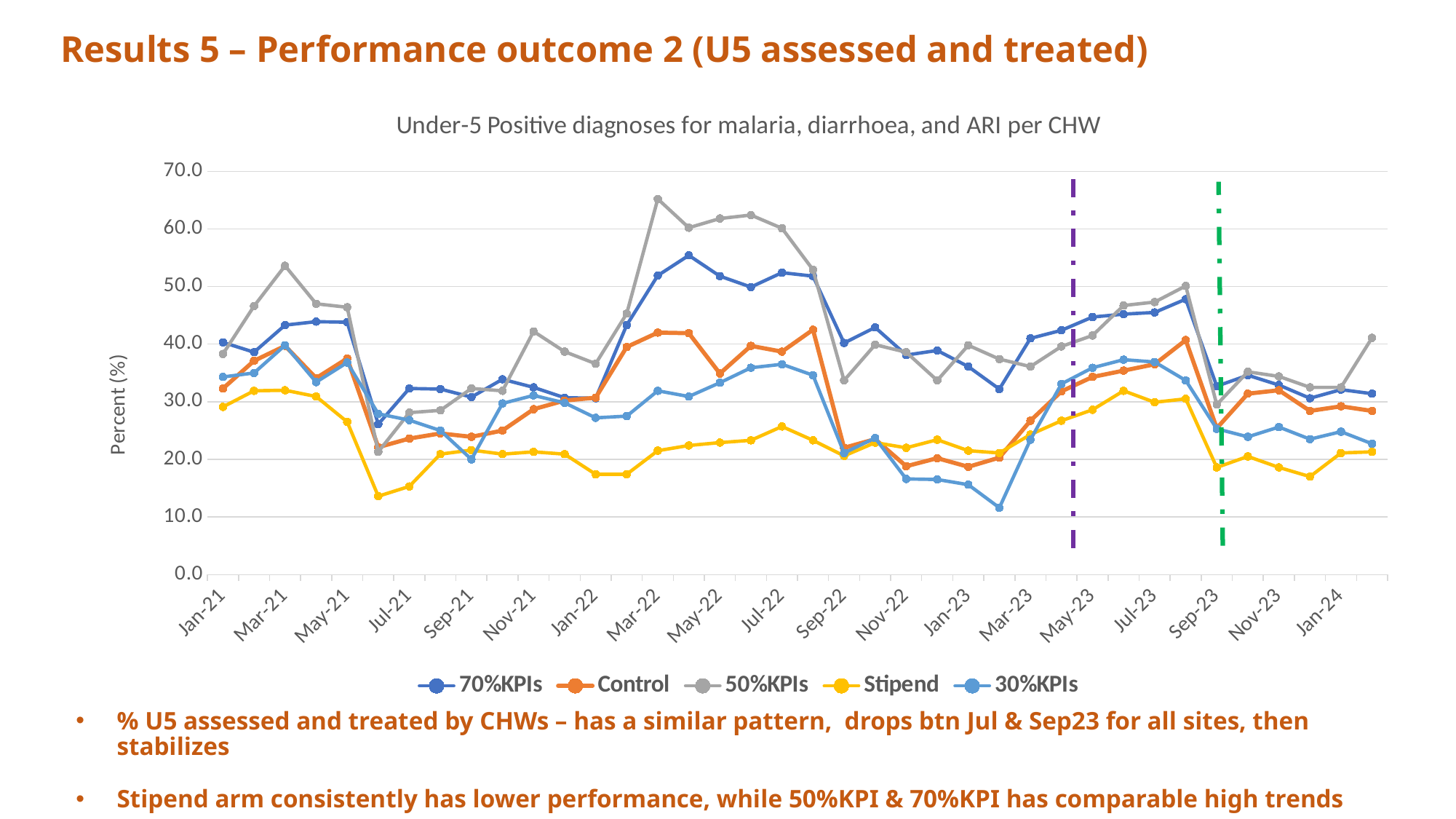

# Results 5 – Performance outcome 2 (U5 assessed and treated)
### Chart: Under-5 Positive diagnoses for malaria, diarrhoea, and ARI per CHW
| Category | 70%KPIs | Control | 50%KPIs | Stipend | 30%KPIs |
|---|---|---|---|---|---|
| 44197 | 40.2999992370605 | 32.2999992370605 | 38.2999992370605 | 29.1000003814697 | 34.2999992370605 |
| 44228 | 38.5999984741211 | 37.0999984741211 | 46.5999984741211 | 31.8999996185303 | 35.0 |
| 44256 | 43.2999992370605 | 39.7000007629395 | 53.5999984741211 | 32.0 | 39.7999992370605 |
| 44287 | 43.9000015258789 | 34.0999984741211 | 47.0 | 30.8999996185303 | 33.4000015258789 |
| 44317 | 43.7999992370605 | 37.5 | 46.4000015258789 | 26.5 | 36.7999992370605 |
| 44348 | 26.1000003814697 | 22.1000003814697 | 21.2999992370606 | 13.6000003814697 | 27.8999996185303 |
| 44378 | 32.2999992370605 | 23.6000003814697 | 28.1000003814697 | 15.3000001907349 | 26.7999992370606 |
| 44409 | 32.2000007629395 | 24.5 | 28.5 | 20.8999996185303 | 25.0 |
| 44440 | 30.7999992370606 | 23.8999996185303 | 32.2999992370605 | 21.6000003814697 | 20.0 |
| 44470 | 33.9000015258789 | 25.0 | 31.8999996185303 | 20.8999996185303 | 29.7000007629394 |
| 44501 | 32.5 | 28.7000007629394 | 42.2000007629395 | 21.2999992370606 | 31.1000003814697 |
| 44531 | 30.70000076293945 | 30.20000076293945 | 38.70000076293945 | 20.89999961853027 | 29.79999923706055 |
| 44562 | 30.60000038146973 | 30.70000076293945 | 36.59999847412109 | 17.39999961853027 | 27.20000076293945 |
| 44593 | 43.29999923706055 | 39.5 | 45.29999923706055 | 17.39999961853027 | 27.5 |
| 44621 | 51.90000152587891 | 42.0 | 65.19999694824219 | 21.5 | 31.89999961853027 |
| 44652 | 55.4 | 41.9 | 60.2 | 22.4 | 30.9 |
| 44682 | 51.8 | 34.9 | 61.8 | 22.9 | 33.3 |
| 44713 | 49.9 | 39.7 | 62.4 | 23.3 | 35.9 |
| 44743 | 52.4 | 38.7 | 60.1 | 25.7 | 36.5 |
| 44774 | 51.8 | 42.5 | 52.9 | 23.3 | 34.6 |
| 44805 | 40.2 | 22.0 | 33.7 | 20.6 | 21.1 |
| 44835 | 42.9 | 23.5 | 39.9 | 22.9 | 23.7 |
| 44866 | 38.1 | 18.8 | 38.6 | 22.0 | 16.6 |
| 44896 | 38.9 | 20.2 | 33.7 | 23.4 | 16.5 |
| 44927 | 36.1 | 18.7 | 39.8 | 21.5 | 15.6 |
| 44958 | 32.2 | 20.3 | 37.4 | 21.1 | 11.6 |
| 44986 | 41.0 | 26.7 | 36.1 | 24.3 | 23.4 |
| 45017 | 42.4 | 31.8 | 39.6 | 26.7 | 33.1 |
| 45047 | 44.7 | 34.3 | 41.5 | 28.6 | 35.9 |
| 45078 | 45.2 | 35.4 | 46.7 | 31.9 | 37.3 |
| 45108 | 45.5 | 36.5 | 47.3 | 29.9 | 36.9 |
| 45139 | 47.8 | 40.7 | 50.1 | 30.5 | 33.7 |
| 45170 | 32.7 | 25.4 | 29.5 | 18.6 | 25.3 |
| 45200 | 34.6 | 31.4 | 35.2 | 20.5 | 23.9 |
| 45231 | 32.9 | 32.0 | 34.4 | 18.6 | 25.6 |
| 45261 | 30.6 | 28.4 | 32.5 | 17.0 | 23.5 |
| 45292 | 32.1 | 29.2 | 32.5 | 21.1 | 24.8 |
| 45323 | 31.4 | 28.4 | 41.1 | 21.3 | 22.7 |% U5 assessed and treated by CHWs – has a similar pattern, drops btn Jul & Sep23 for all sites, then stabilizes
Stipend arm consistently has lower performance, while 50%KPI & 70%KPI has comparable high trends

## Slide 25
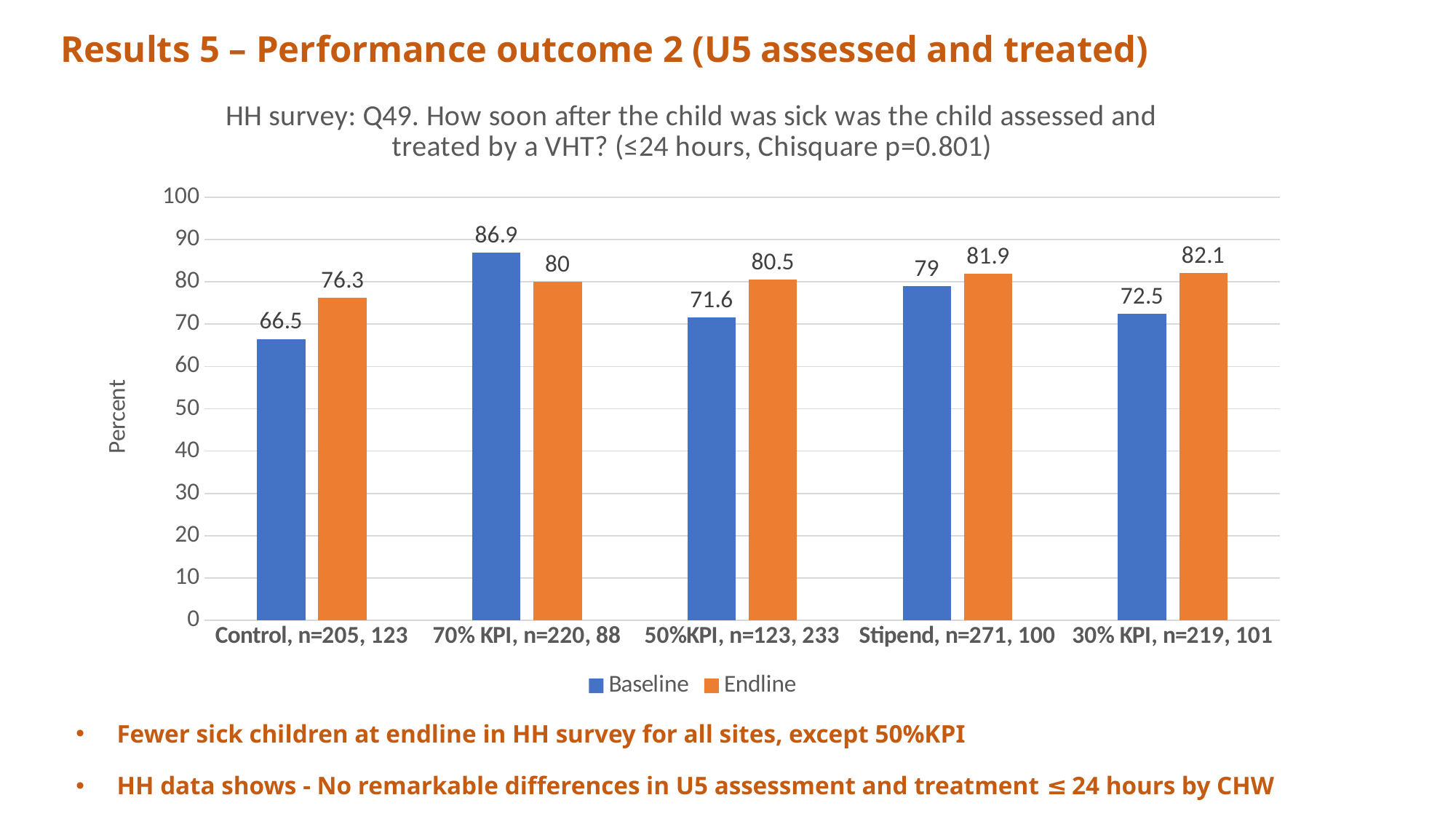

# Results 5 – Performance outcome 2 (U5 assessed and treated)
### Chart: HH survey: Q49. How soon after the child was sick was the child assessed and treated by a VHT? (≤24 hours, Chisquare p=0.801)
| Category | Baseline | Endline |
|---|---|---|
| Control, n=205, 123 | 66.5 | 76.3 |
| 70% KPI, n=220, 88 | 86.9 | 80.0 |
| 50%KPI, n=123, 233 | 71.6 | 80.5 |
| Stipend, n=271, 100 | 79.0 | 81.9 |
| 30% KPI, n=219, 101 | 72.5 | 82.1 |Fewer sick children at endline in HH survey for all sites, except 50%KPI
HH data shows - No remarkable differences in U5 assessment and treatment ≤ 24 hours by CHW

## Slide 26
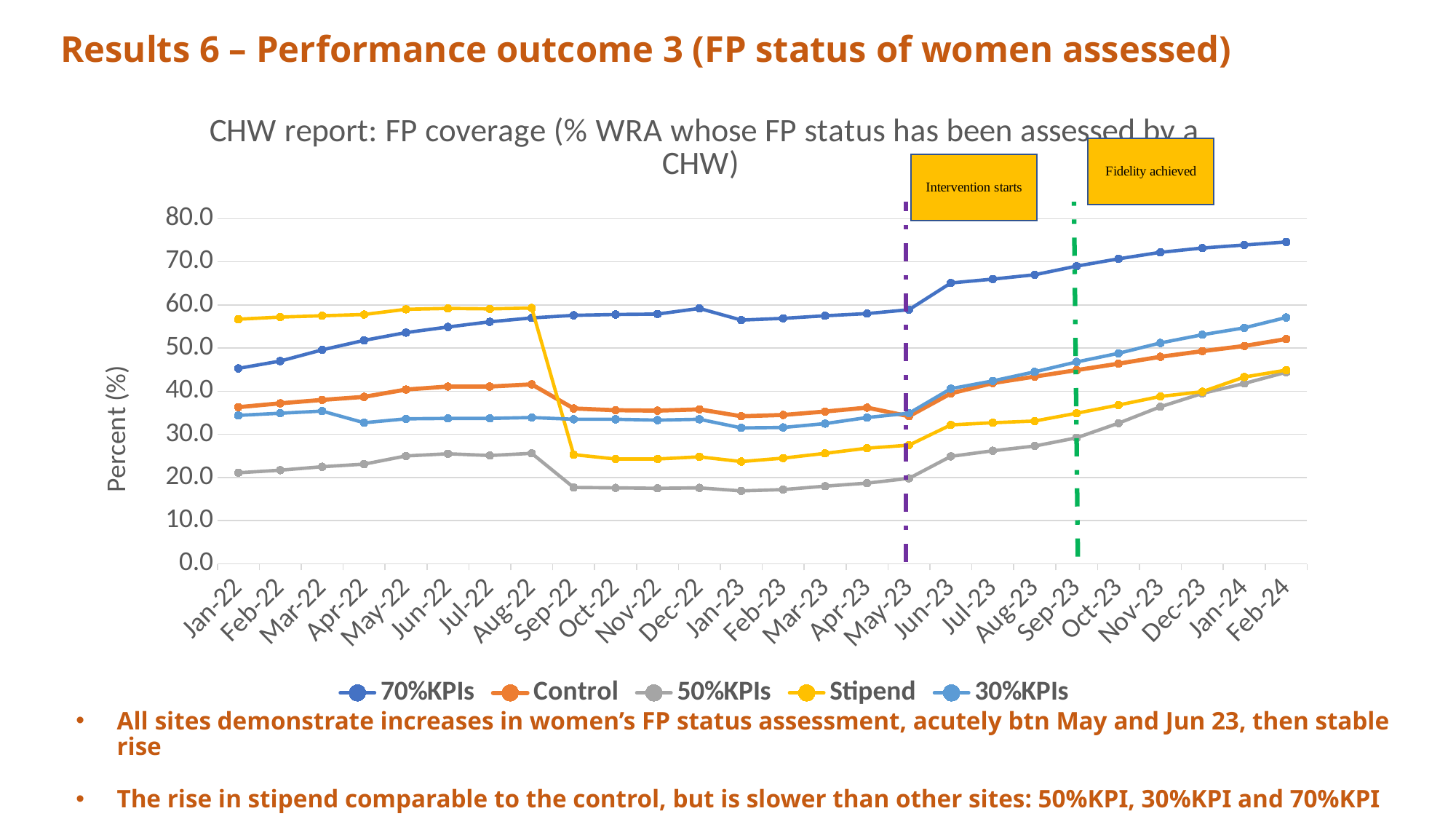

# Results 6 – Performance outcome 3 (FP status of women assessed)
### Chart: CHW report: FP coverage (% WRA whose FP status has been assessed by a CHW)
| Category | 70%KPIs | Control | 50%KPIs | Stipend | 30%KPIs |
|---|---|---|---|---|---|
| 44562 | 45.3 | 36.3 | 21.1 | 56.7 | 34.4 |
| 44593 | 47.0 | 37.2 | 21.7 | 57.2 | 34.9 |
| 44621 | 49.6 | 38.0 | 22.5 | 57.5 | 35.4 |
| 44652 | 51.8 | 38.7 | 23.1 | 57.8 | 32.7 |
| 44682 | 53.6 | 40.4 | 25.0 | 59.0 | 33.6 |
| 44713 | 54.9 | 41.1 | 25.5 | 59.2 | 33.7 |
| 44743 | 56.1 | 41.1 | 25.1 | 59.1 | 33.7 |
| 44774 | 57.0 | 41.6 | 25.6 | 59.3 | 33.9 |
| 44805 | 57.6 | 36.0 | 17.7 | 25.3 | 33.5 |
| 44835 | 57.8 | 35.6 | 17.6 | 24.3 | 33.5 |
| 44866 | 57.9 | 35.5 | 17.5 | 24.3 | 33.3 |
| 44896 | 59.2 | 35.8 | 17.6 | 24.8 | 33.5 |
| 44927 | 56.5 | 34.2 | 16.9 | 23.7 | 31.5 |
| 44958 | 56.9 | 34.5 | 17.2 | 24.5 | 31.6 |
| 44986 | 57.5 | 35.3 | 18.0 | 25.6 | 32.5 |
| 45017 | 58.0 | 36.2 | 18.7 | 26.8 | 33.9 |
| 45047 | 58.9 | 34.2 | 19.8 | 27.5 | 34.9 |
| 45078 | 65.1 | 39.5 | 24.9 | 32.2 | 40.6 |
| 45108 | 66.0 | 41.9 | 26.2 | 32.7 | 42.4 |
| 45139 | 67.0 | 43.4 | 27.3 | 33.1 | 44.5 |
| 45170 | 69.0 | 44.9 | 29.2 | 34.9 | 46.8 |
| 45200 | 70.7 | 46.4 | 32.6 | 36.8 | 48.8 |
| 45231 | 72.2 | 48.0 | 36.4 | 38.8 | 51.2 |
| 45261 | 73.2 | 49.3 | 39.5 | 39.9 | 53.1 |
| 45292 | 73.9 | 50.5 | 41.8 | 43.3 | 54.7 |
| 45323 | 74.6 | 52.1 | 44.4 | 44.9 | 57.1 |All sites demonstrate increases in women’s FP status assessment, acutely btn May and Jun 23, then stable rise
The rise in stipend comparable to the control, but is slower than other sites: 50%KPI, 30%KPI and 70%KPI

## Slide 27
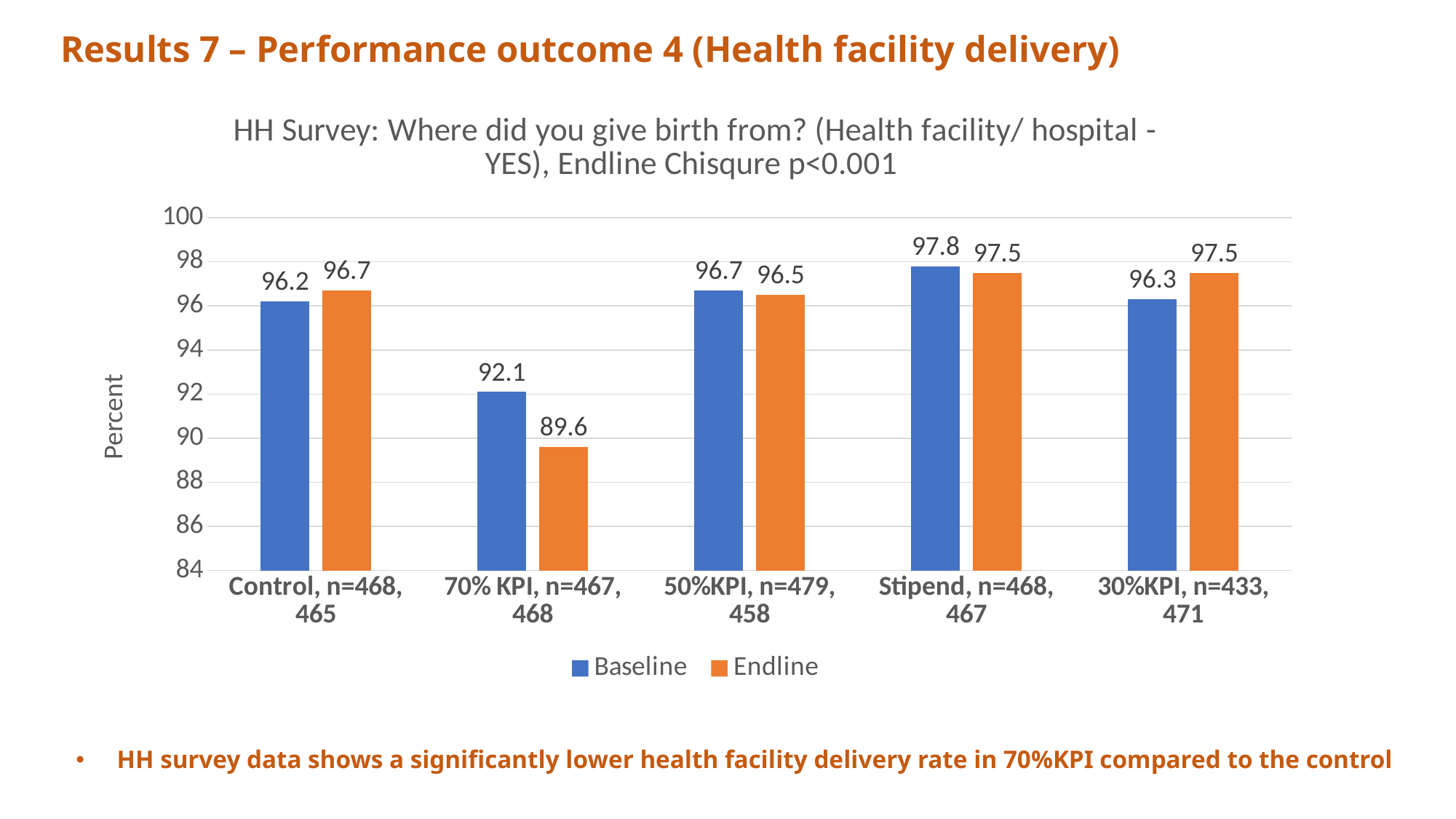

# Results 7 – Performance outcome 4 (Health facility delivery)
### Chart: HH Survey: Where did you give birth from? (Health facility/ hospital - YES), Endline Chisqure p<0.001
| Category | Baseline | Endline |
|---|---|---|
| Control, n=468, 465 | 96.2 | 96.7 |
| 70% KPI, n=467, 468 | 92.1 | 89.6 |
| 50%KPI, n=479, 458 | 96.7 | 96.5 |
| Stipend, n=468, 467 | 97.8 | 97.5 |
| 30%KPI, n=433, 471 | 96.3 | 97.5 |HH survey data shows a significantly lower health facility delivery rate in 70%KPI compared to the control

## Slide 28
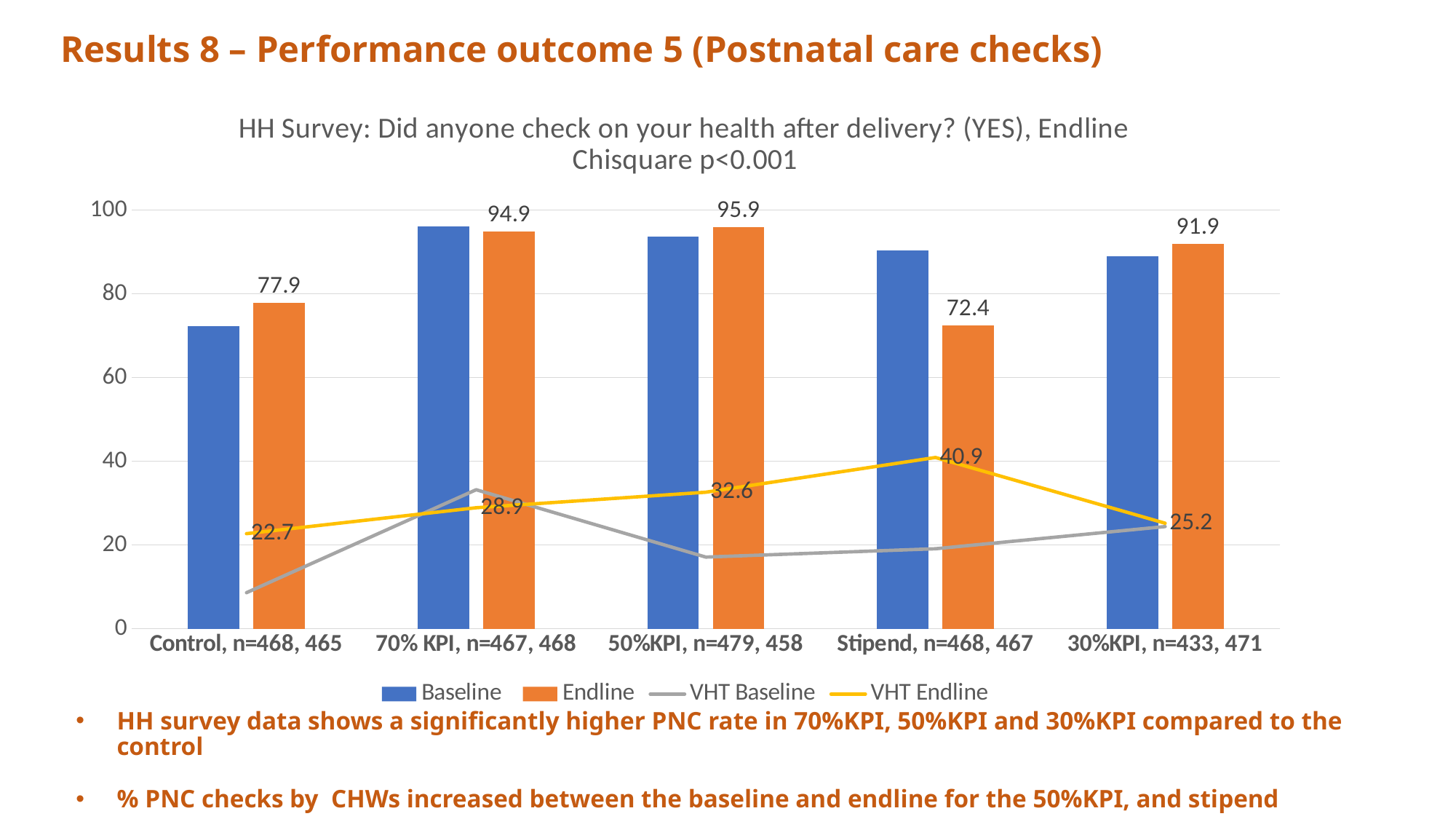

# Results 8 – Performance outcome 5 (Postnatal care checks)
### Chart: HH Survey: Did anyone check on your health after delivery? (YES), Endline Chisquare p<0.001
| Category | Baseline | Endline | VHT Baseline | VHT Endline |
|---|---|---|---|---|
| Control, n=468, 465 | 72.2 | 77.9 | 8.6 | 22.7 |
| 70% KPI, n=467, 468 | 96.2 | 94.9 | 33.2 | 28.9 |
| 50%KPI, n=479, 458 | 93.7 | 95.9 | 17.1 | 32.6 |
| Stipend, n=468, 467 | 90.4 | 72.4 | 19.1 | 40.9 |
| 30%KPI, n=433, 471 | 88.9 | 91.9 | 24.4 | 25.2 |HH survey data shows a significantly higher PNC rate in 70%KPI, 50%KPI and 30%KPI compared to the control
% PNC checks by CHWs increased between the baseline and endline for the 50%KPI, and stipend

## Slide 29
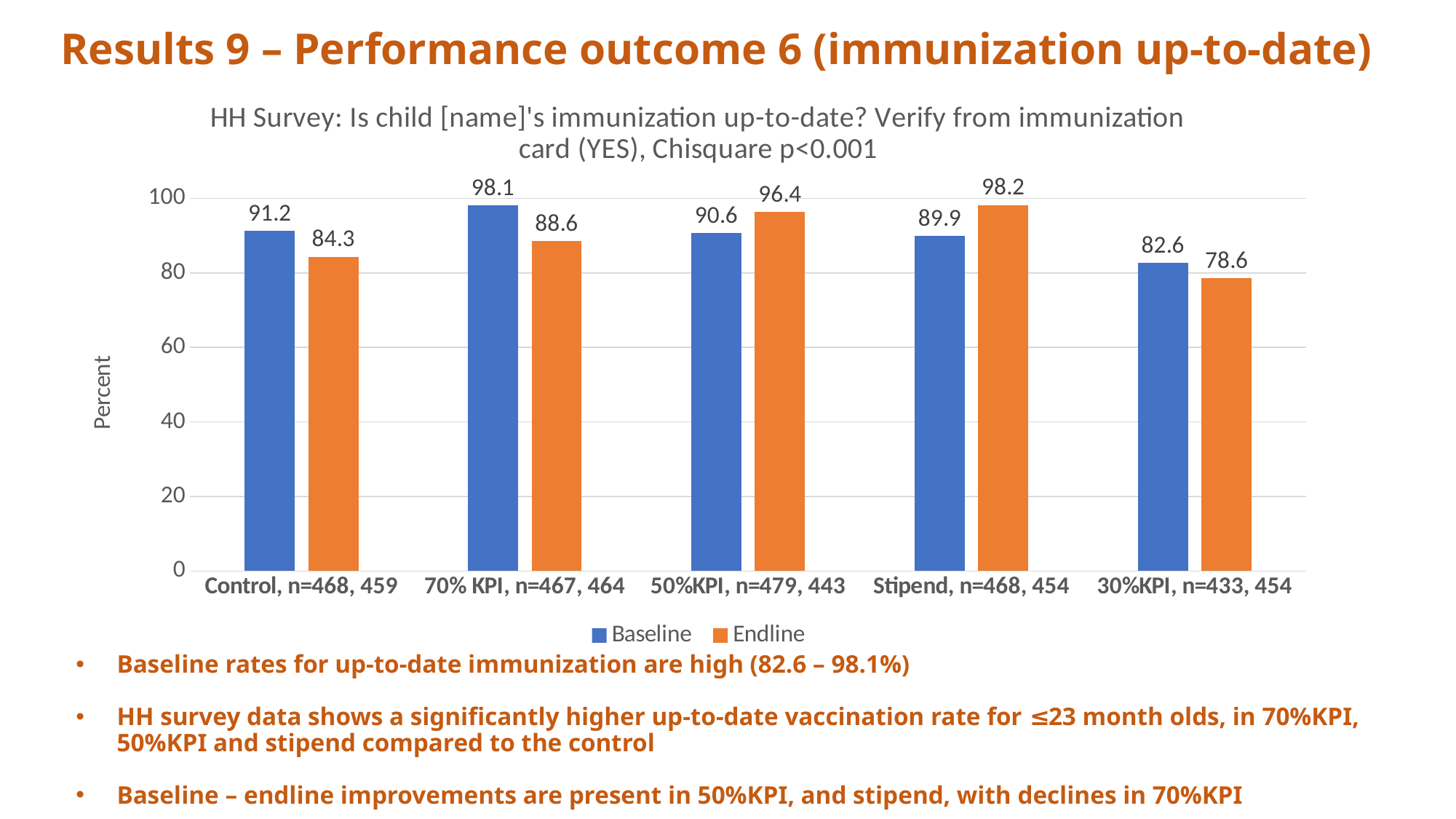

# Results 9 – Performance outcome 6 (immunization up-to-date)
### Chart: HH Survey: Is child [name]'s immunization up-to-date? Verify from immunization card (YES), Chisquare p<0.001
| Category | Baseline | Endline |
|---|---|---|
| Control, n=468, 459 | 91.2 | 84.3 |
| 70% KPI, n=467, 464 | 98.1 | 88.6 |
| 50%KPI, n=479, 443 | 90.6 | 96.4 |
| Stipend, n=468, 454 | 89.9 | 98.2 |
| 30%KPI, n=433, 454 | 82.6 | 78.6 |Baseline rates for up-to-date immunization are high (82.6 – 98.1%)
HH survey data shows a significantly higher up-to-date vaccination rate for ≤23 month olds, in 70%KPI, 50%KPI and stipend compared to the control
Baseline – endline improvements are present in 50%KPI, and stipend, with declines in 70%KPI

## Slide 30
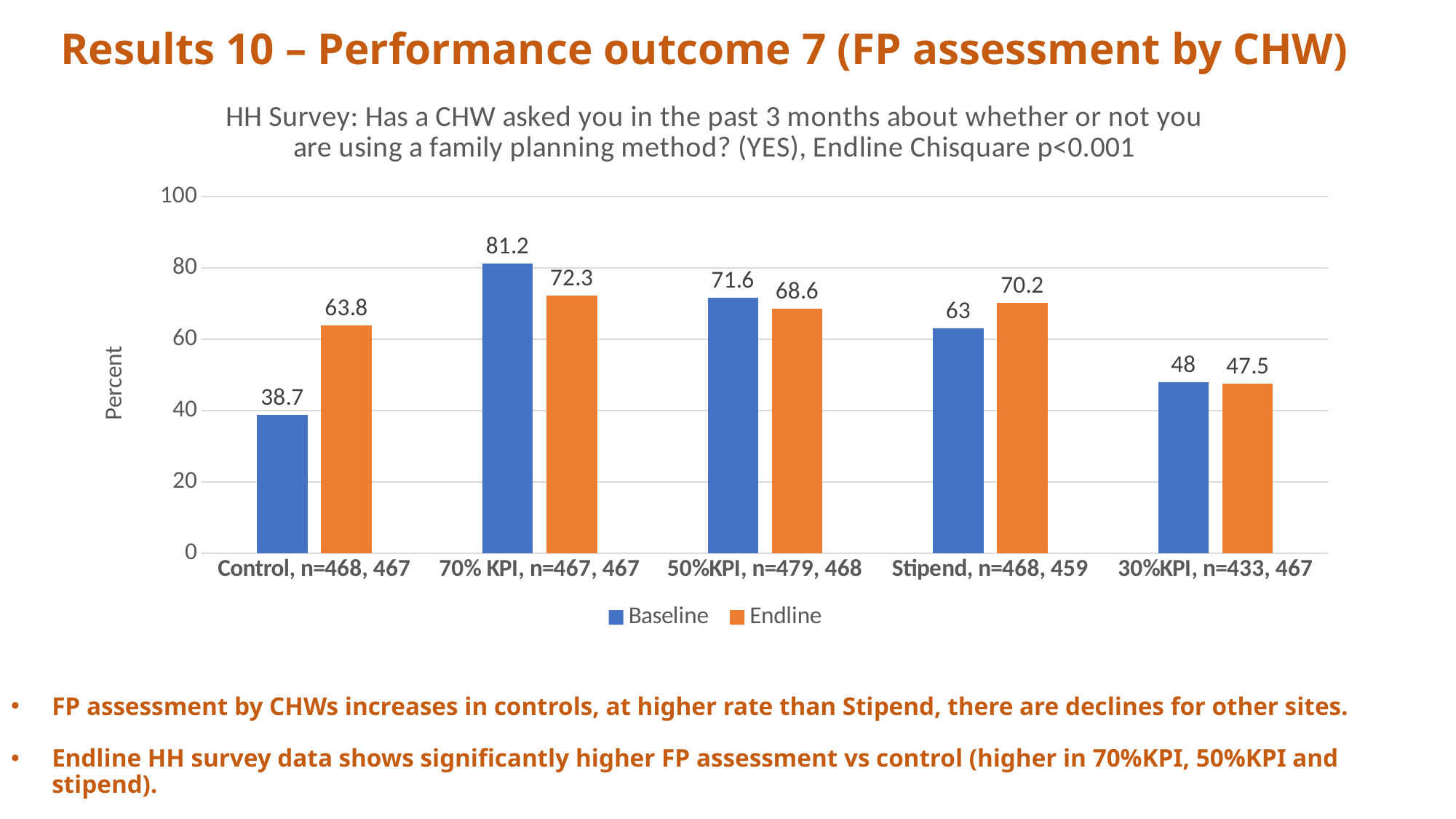

# Results 10 – Performance outcome 7 (FP assessment by CHW)
### Chart: HH Survey: Has a CHW asked you in the past 3 months about whether or not you are using a family planning method? (YES), Endline Chisquare p<0.001
| Category | Baseline | Endline |
|---|---|---|
| Control, n=468, 467 | 38.7 | 63.8 |
| 70% KPI, n=467, 467 | 81.2 | 72.3 |
| 50%KPI, n=479, 468 | 71.6 | 68.6 |
| Stipend, n=468, 459 | 63.0 | 70.2 |
| 30%KPI, n=433, 467 | 48.0 | 47.5 |FP assessment by CHWs increases in controls, at higher rate than Stipend, there are declines for other sites.
Endline HH survey data shows significantly higher FP assessment vs control (higher in 70%KPI, 50%KPI and stipend).

## Slide 31
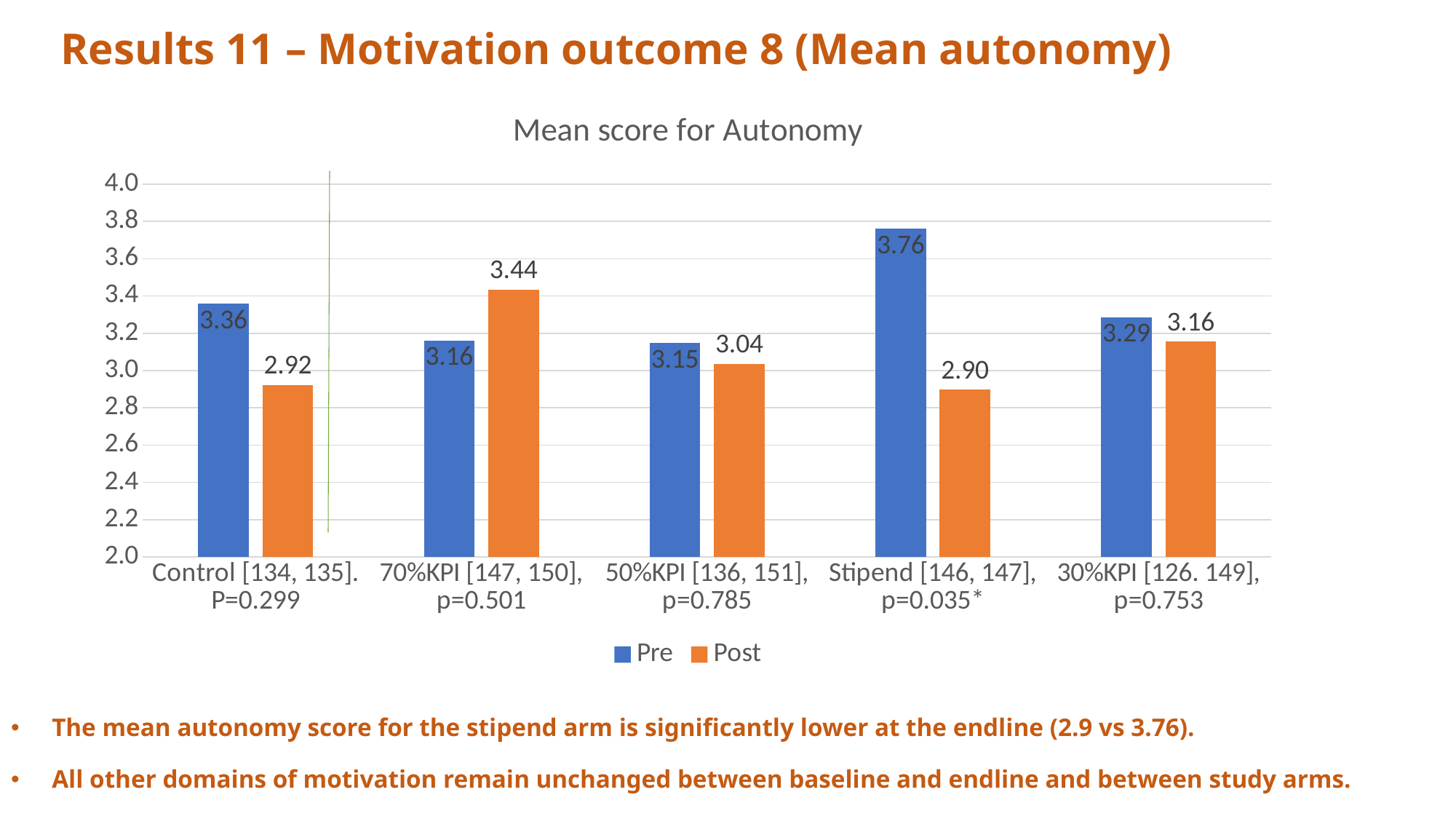

# Results 11 – Motivation outcome 8 (Mean autonomy)
### Chart: Mean score for Autonomy
| Category | Pre | Post |
|---|---|---|
| Control [134, 135]. P=0.299 | 3.358333333333333 | 2.921666666666667 |
| 70%KPI [147, 150], p=0.501 | 3.1616666666666666 | 3.435 |
| 50%KPI [136, 151], p=0.785 | 3.1466666666666665 | 3.0366666666666666 |
| Stipend [146, 147], p=0.035* | 3.76 | 2.8966666666666665 |
| 30%KPI [126. 149], p=0.753 | 3.2866666666666666 | 3.155 |The mean autonomy score for the stipend arm is significantly lower at the endline (2.9 vs 3.76).
All other domains of motivation remain unchanged between baseline and endline and between study arms.

## Slide 32
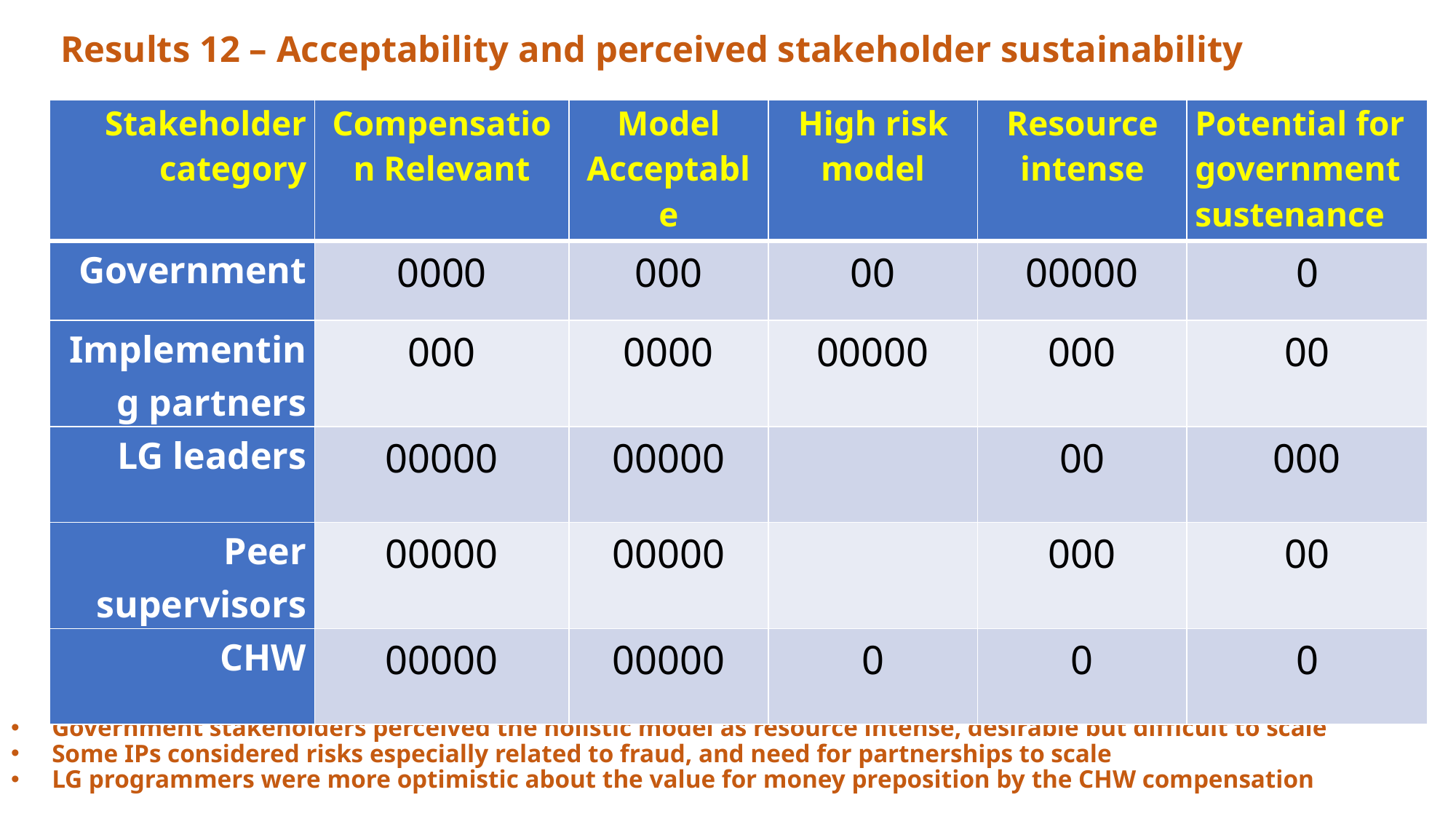

# Results 12 – Acceptability and perceived stakeholder sustainability
| Stakeholder category | Compensation Relevant | Model Acceptable | High risk model | Resource intense | Potential for government sustenance |
| --- | --- | --- | --- | --- | --- |
| Government | 0000 | 000 | 00 | 00000 | 0 |
| Implementing partners | 000 | 0000 | 00000 | 000 | 00 |
| LG leaders | 00000 | 00000 | | 00 | 000 |
| Peer supervisors | 00000 | 00000 | | 000 | 00 |
| CHW | 00000 | 00000 | 0 | 0 | 0 |
Government stakeholders perceived the holistic model as resource intense, desirable but difficult to scale
Some IPs considered risks especially related to fraud, and need for partnerships to scale
LG programmers were more optimistic about the value for money preposition by the CHW compensation

## Slide 33
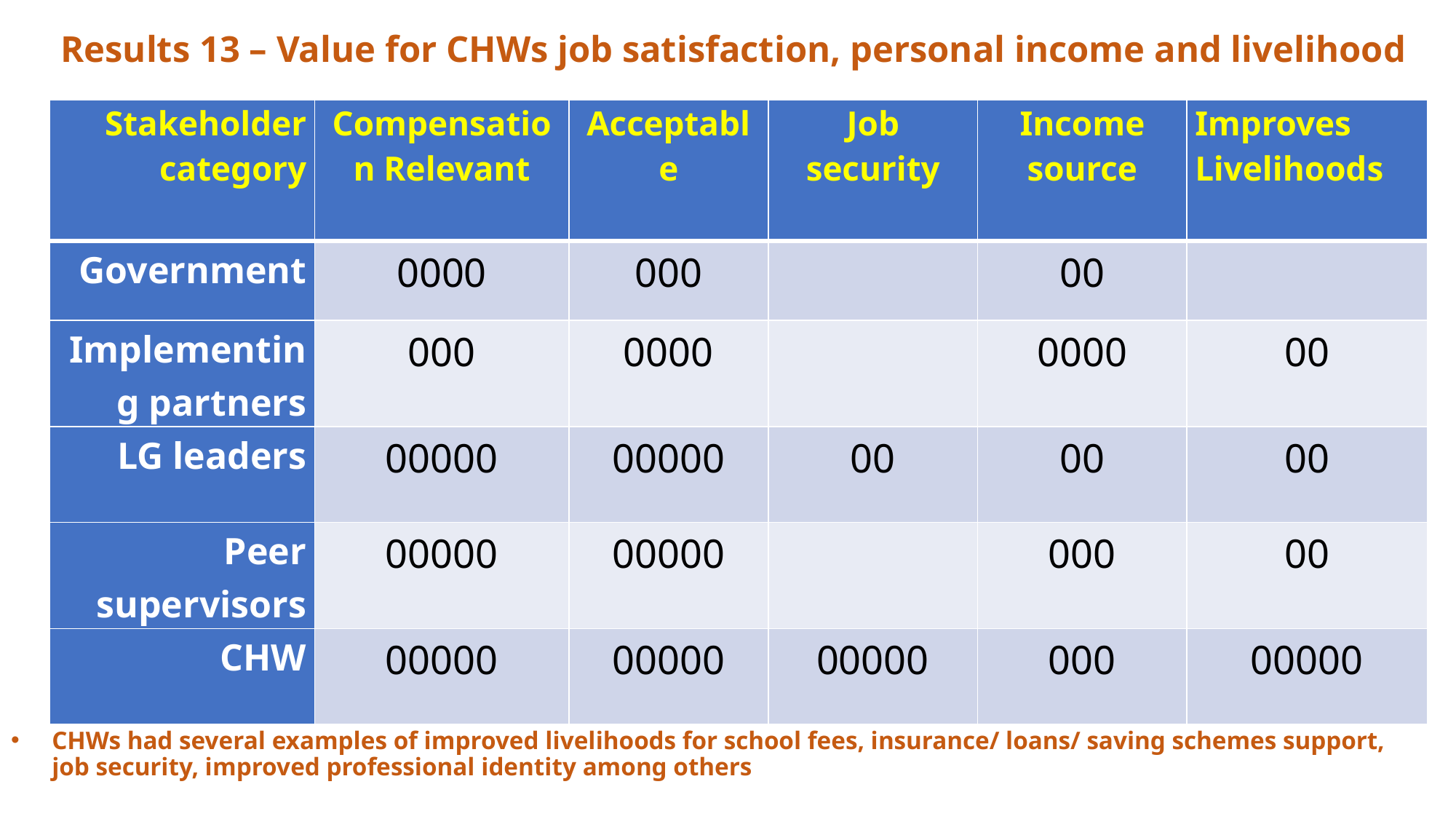

# Results 13 – Value for CHWs job satisfaction, personal income and livelihood
| Stakeholder category | Compensation Relevant | Acceptable | Job security | Income source | Improves Livelihoods |
| --- | --- | --- | --- | --- | --- |
| Government | 0000 | 000 | | 00 | |
| Implementing partners | 000 | 0000 | | 0000 | 00 |
| LG leaders | 00000 | 00000 | 00 | 00 | 00 |
| Peer supervisors | 00000 | 00000 | | 000 | 00 |
| CHW | 00000 | 00000 | 00000 | 000 | 00000 |
CHWs had several examples of improved livelihoods for school fees, insurance/ loans/ saving schemes support, job security, improved professional identity among others

## Slide 34
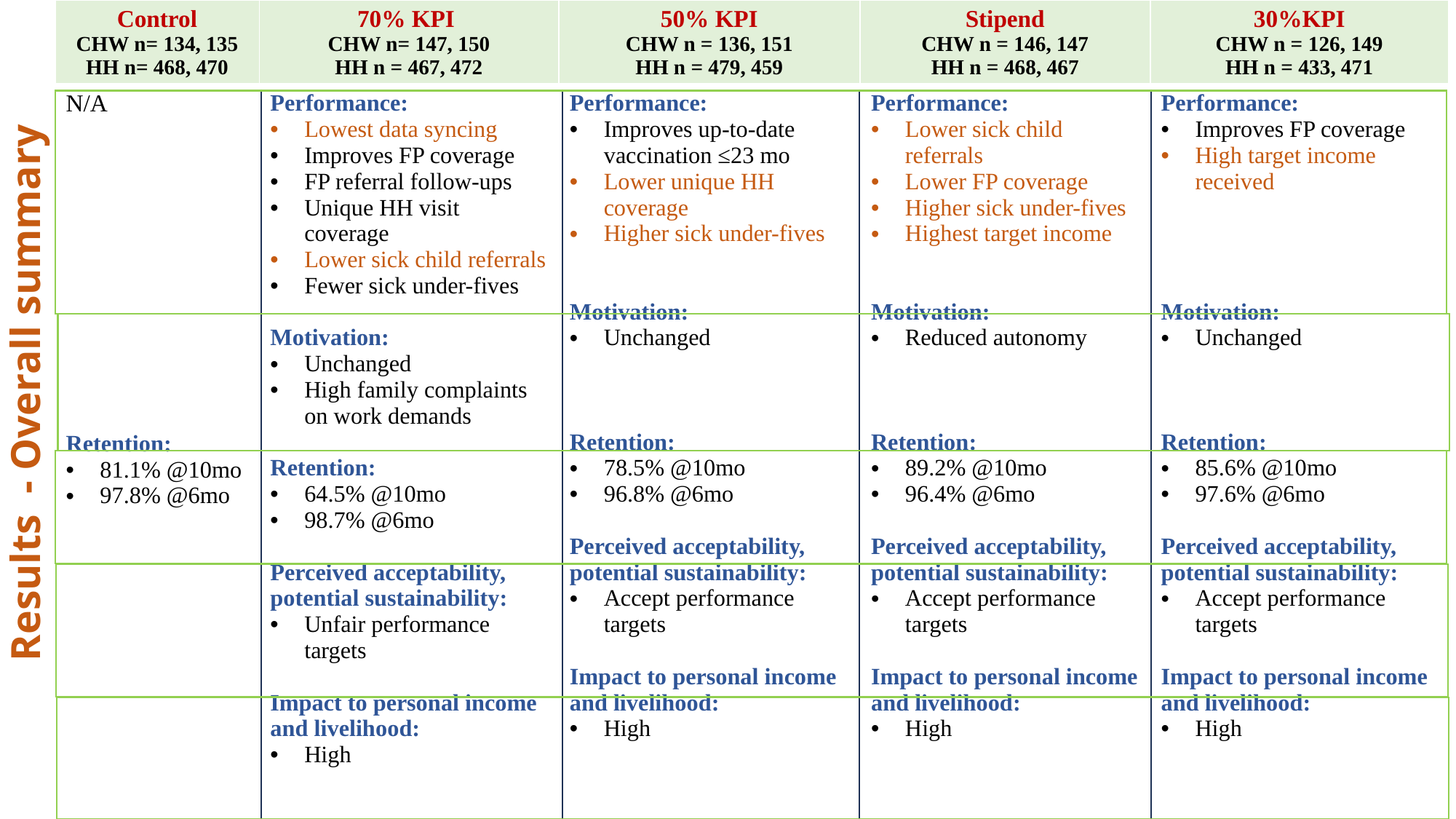

| Control CHW n= 134, 135 HH n= 468, 470 | 70% KPI CHW n= 147, 150 HH n = 467, 472 | 50% KPI CHW n = 136, 151 HH n = 479, 459 | Stipend CHW n = 146, 147 HH n = 468, 467 | 30%KPI CHW n = 126, 149 HH n = 433, 471 |
| --- | --- | --- | --- | --- |
| N/A Retention: 81.1% @10mo 97.8% @6mo | Performance: Lowest data syncing Improves FP coverage FP referral follow-ups Unique HH visit coverage Lower sick child referrals Fewer sick under-fives Motivation: Unchanged High family complaints on work demands Retention: 64.5% @10mo 98.7% @6mo Perceived acceptability, potential sustainability: Unfair performance targets Impact to personal income and livelihood: High | Performance: Improves up-to-date vaccination ≤23 mo Lower unique HH coverage Higher sick under-fives Motivation: Unchanged Retention: 78.5% @10mo 96.8% @6mo Perceived acceptability, potential sustainability: Accept performance targets Impact to personal income and livelihood: High | Performance: Lower sick child referrals Lower FP coverage Higher sick under-fives Highest target income Motivation: Reduced autonomy Retention: 89.2% @10mo 96.4% @6mo Perceived acceptability, potential sustainability: Accept performance targets Impact to personal income and livelihood: High | Performance: Improves FP coverage High target income received Motivation: Unchanged Retention: 85.6% @10mo 97.6% @6mo Perceived acceptability, potential sustainability: Accept performance targets Impact to personal income and livelihood: High |
# Results - Overall summary

## Slide 35
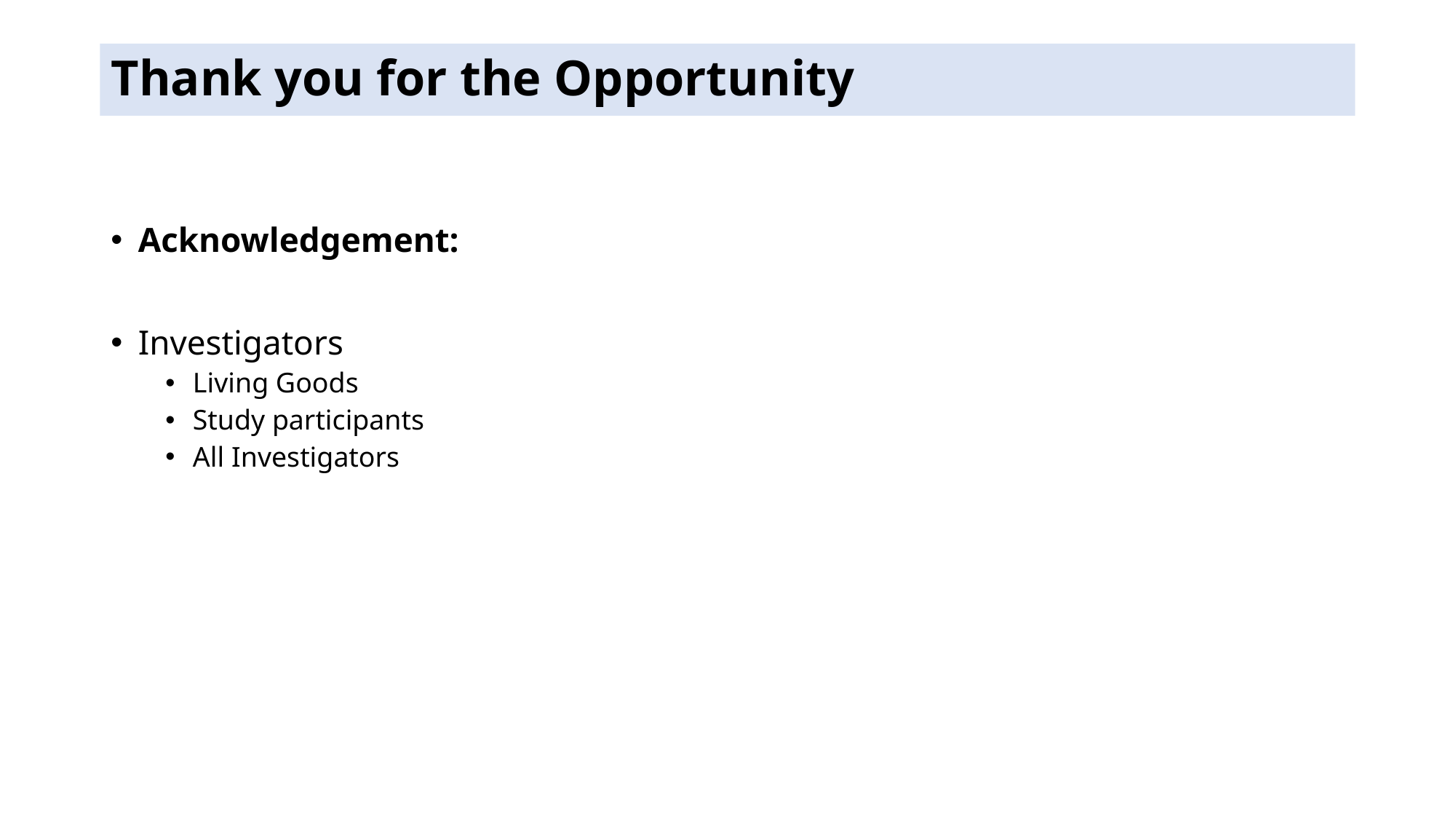

# Thank you for the Opportunity
Acknowledgement:
Investigators
Living Goods
Study participants
All Investigators
