# Supplementary material for: Effects of financial compensation structures on community health worker performance, motivation, and retention: evidence from a multi-arm quasi-experiment in Uganda
Source: Front Health Serv. 2025 Dec 11;5:1687782. doi: 10.3389/frhs.2025.1687782 (PMC12738377; doi:10.3389/frhs.2025.1687782)
Supplement: Supplementary file 2 [file Datasheet1.docx]

**Appendix B**

**Results 3 – Performance processes 1 (data syncing)**

Overall data syncing rose across sites between May and Sep23, then begun to decline - ? unclear why?

70%KPI arm had declining data syncing after Sep23 - ? Is this related to lower motivation/ supervision/ technological issues?

Results 3 – Performance processes 2 (earn income targets)

Overall income earning rises between June and Oct23, then stabilizes – likely threshold of performance reached

- **Overall income earning rises between June and Oct23, then stabilizes – likely threshold of performance reached**
- **Stipend arm has highest earning of income targets, but variability is high between study arms**

Stipend arm has highest earning of income targets, but variability is high between study arms

**Results 4 – Performance outcome 1 (unique HH visits)**

- **Unique HH visits appear to remain stable across most arms**
- **The 30%KPI arm has steady rise in % unique HH visit**

**Results 5 – Performance outcome 2 (U5 assessed and treated)**

- **% U5 assessed and treated by CHWs – has a similar pattern, drops btn Jul & Sep23 for all sites, then stabilizes**
- **Stipend arm consistently has lower performance, while 50%KPI & 70%KPI has comparable high trends**
